# Three unrelated protease inhibitors enhance accumulation of pharmaceutical recombinant proteins in *Nicotiana benthamiana*

**DOI:** 10.1111/pbi.12916

**Published:** 2018-05-24

**Authors:** Friederike Grosse‐Holz, Luisa Madeira, Muhammad Awais Zahid, Molly Songer, Jiorgos Kourelis, Mary Fesenko, Sabrina Ninck, Farnusch Kaschani, Markus Kaiser, Renier A.L. van der Hoorn

**Affiliations:** ^1^ Plant Chemetics Laboratory Department of Plant Sciences University of Oxford Oxford UK; ^2^ Chemische Biologie Zentrum für Medizinische Biotechnologie Fakultät für Biologie Universität Duisburg‐Essen Universitätsstr Essen Germany

**Keywords:** *Agrobacterium tumefaciens*, transient expression, agroinfiltration, *Nicotiana benthamiana*, activity‐based protein profiling, protease inhibitor

## Abstract

Agroinfiltrated *Nicotiana benthamiana* is a flexible and scalable platform for recombinant protein (RP) production, but its great potential is hampered by plant proteases that degrade RPs. Here, we tested 29 candidate protease inhibitors (PIs) in agroinfiltrated *N. benthamiana* leaves for enhancing accumulation of three unrelated RPs: glycoenzyme α‐Galactosidase; glycohormone erythropoietin (EPO); and IgG antibody VRC01. Of the previously described PIs enhancing RP accumulation, we found only cystatin SlCYS8 to be effective. We identified three additional new, unrelated PIs that enhance RP accumulation: *N. benthamiana* NbPR4, NbPot1 and human HsTIMP, which have been reported to inhibit cysteine, serine and metalloproteases, respectively. Remarkably, accumulation of all three RPs is enhanced by each PI similarly, suggesting that the mechanism of degradation of unrelated RPs follows a common pathway. Inhibitory functions HsTIMP and SlCYS8 are required to enhance RP accumulation, suggesting that their target proteases may degrade RPs. Different PIs additively enhance RP accumulation, but the effect of each PI is dose‐dependent. Activity‐based protein profiling (ABPP) revealed that the activities of papain‐like Cys proteases (PLCPs), Ser hydrolases (SHs) or vacuolar processing enzymes (VPEs) in leaves are unaffected upon expression of the new PIs, whereas SlCYS8 expression specifically suppresses PLCP activity only. Quantitative proteomics indicates that the three new PIs affect agroinfiltrated tissues similarly and that they all increase immune responses. NbPR4, NbPot1 and HsTIMP can be used to study plant proteases and improve RP accumulation in molecular farming.

## Introduction

Molecular farming, the production of biopharmaceuticals in plants, offers speed, scalability and low risk of contamination with human pathogens when compared to insect or mammalian cell culture systems (Stoger *et al*., 2014). Plant‐made biopharmaceuticals are now a reality, with the first product on the market (Fox, 2012) and more in clinical trials (Lomonossoff and D'Aoust, 2016). To produce biopharmaceuticals, *N. benthamiana* leaves can be genetically modified by infiltration with disarmed *Agrobacterium tumefaciens* (Agrobacterium) carrying gene(s) of interest on the transfer DNA (T‐DNA) of binary plasmid(s) (Bevan, 1984). Agrobacterium delivers the T‐DNA to the plant nucleus, allowing foreign genes to be transiently expressed. Co‐expression of several transgenes is achieved simply by mixing Agrobacterium cultures delivering different transgenes before agroinfiltration. Co‐expression with silencing inhibitor P19 is frequently used to boost protein overexpression by preventing the decline of the transgene transcript levels (Van Der Hoorn *et al*., 2003). Agroinfiltration‐mediated protein expression can now deliver ten million doses of the latest influenza vaccine within 6 weeks *(Pillet et al.,* 2016). Large‐scale agroinfiltration has also been used to produce functional monoclonal antibodies (mAbs, Yusibov *et al*., 2016), including the Ebola‐neutralizing drug ZMapp (Qiu *et al*., 2014). To maximize efficacy and limit immunogenicity, biopharmaceuticals like ZMapp are produced with humanized *N‐*glycans in the secretory pathway of genetically engineered *N. benthamiana* (Castilho *et al*., 2014; Schoberer and Strasser, 2017).

A bottleneck on the road to commercialization of agroinfiltration for molecular farming is the relatively low yields of plant‐produced recombinant proteins (RPs): 15–200 mg of monoclonal antibody (mAb) or <1 g virus‐like particles per kg fresh leaf (Lomonossoff and D'Aoust, 2016; Yusibov *et al*., 2016). Both yield and purity of RPs are hampered by proteolytic degradation (Donini *et al*., 2015; Doran, 2006; Hehle *et al*., 2015; Niemer *et al*., 2014), which can occur in the extracellular space (Hehle *et al*., 2011) and act on RPs as they pass through the secretory pathway for glycosylation. Several *N. benthamiana* papain‐like Cys proteases (PLCPs) can degrade RPs *in vitro* (Paireder *et al*., 2016, 2017), but the proteases degrading RPs in agroinfiltrated leaves remain to be identified.

The *N. benthamiana* protease repertoire is large and diverse. We recently described transcripts corresponding to 975 putative proteases of all catalytic classes present in agroinfiltrated leaves. We also detected peptides corresponding to 196 proteases in the extracellular space (Grosse‐Holz *et al*., 2018). RPs are thus exposed to a large, complex proteolytic network in agroinfiltrated *N. benthamiana*. Past attempts to prevent RP degradation include the use of stabilizing agents, protease gene knockdown, subcellular targeting, fusion proteins and protease inhibitor co‐expression (Mandal *et al*., 2016). However, no ‘magic bullet’ against degradation has been identified.

Among the protease activity depletion approaches, protease inhibitor (PI) co‐expression is promising for three reasons. First, many PIs can inhibit several enzymes, often from different families, overcoming protease redundancy (Grosse‐Holz and van der Hoorn, 2016). Second, PIs can be targeted to the secretory pathway to escort RPs during secretion (Goulet *et al*., 2012; Jutras *et al*., 2016). Third, PI co‐expression by agroinfiltration depletes protease activity only temporally and locally, thus protecting RPs with minimal effects on nonagroinfiltrated tissues.

To date, six PIs were found to enhance RP accumulation (Mandal *et al*., 2016). Most of the PIs that enhanced RP accumulation *in planta* were expressed in stable transgenic plant cells: a Bowman‐Birk Ser protease inhibitor boosted mAb accumulation in *N. tabacum* roots (Komarnytsky *et al*., 2006) and a *N. alata* Ser protease inhibitor (Protease inhibitor II) enhanced accumulation of human granulocyte–macrophage colony stimulating factor (hGM‐CSF) in rice suspension cells (Kim *et al*., 2008). Likewise, transgenic tobacco plants expressing oryzacystatin I produced increased levels of recombinant glutathione reductase (Pillay *et al*., 2012), and tomato cathepsin D inhibitor (SlCDI) has been used to enhance human α_1_‐anti‐chymotrypsin accumulation in transgenic potato leaves (Goulet *et al*., 2010) and boost mAb levels upon transient co‐expression in *N. benthamiana* (Goulet *et al*., 2012). However, effects of transient SlCDI co‐expression on mAb levels seem to depend on plant growth conditions (Robert *et al*., 2013). The tomato Cys protease inhibitor SlCYS8 enhances accumulation of mAbs upon co‐expression in agroinfiltrated *N. benthamiana* (Jutras *et al*., 2016; Robert *et al*., 2013) and can act as a stabilizing fusion partner to increase human α_1_‐anti‐chymotrypsin levels (Sainsbury *et al*., 2013).

Here, we expand the toolbox of protease inhibitors for molecular farming, specifically in agroinfiltrated *N. benthamiana*, through a systematic screen of candidate PIs targeting various classes of *N. benthamiana* proteases. We selected three new PIs that increase levels of three unrelated RPs, separately and in combination. We also investigated suppression of protease activity and identified changes in the total proteome of leaves upon PI overexpression.

## Results

### Selecting candidate protease inhibitors

**Figure 1:**
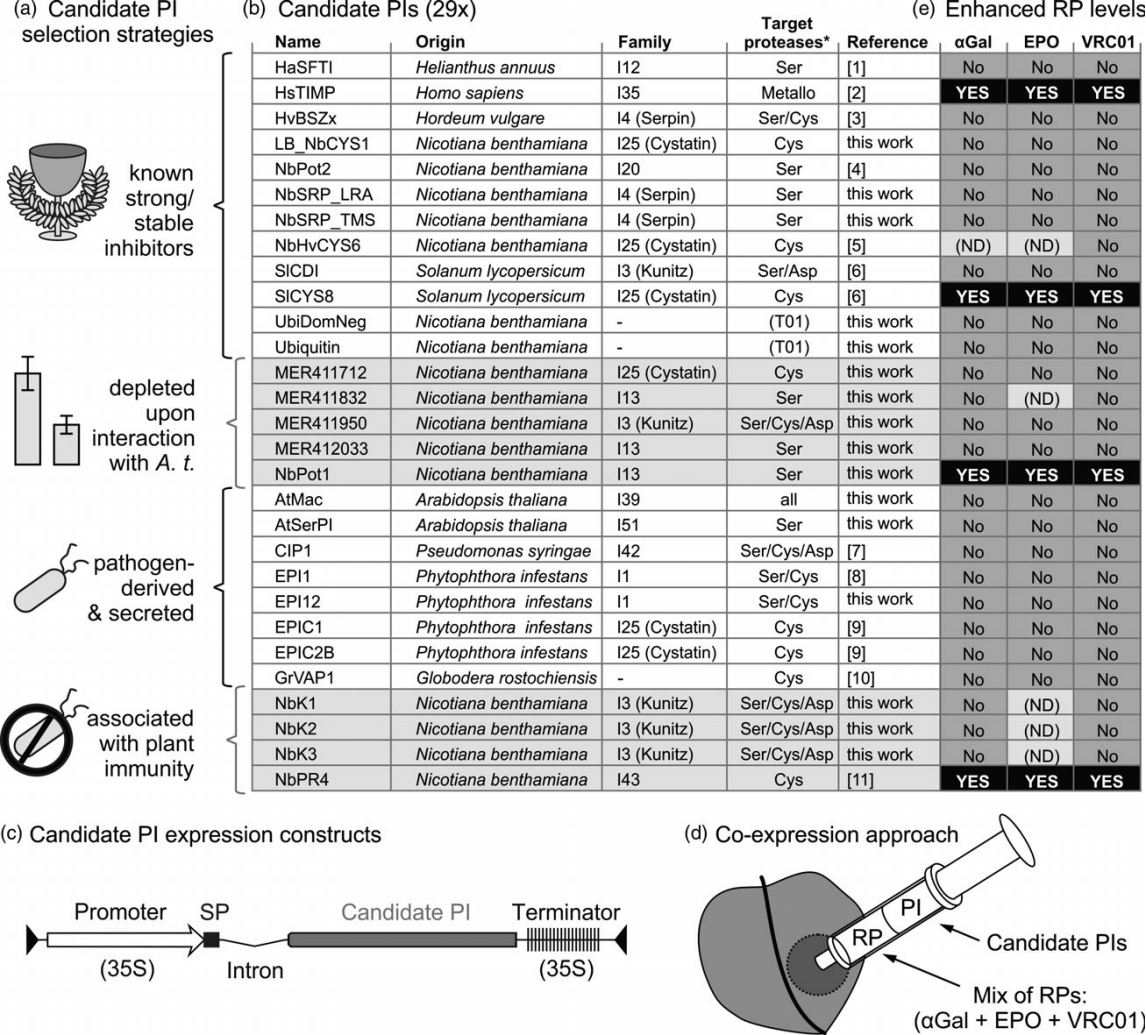
Four of 29 tested candidate protease inhibitors (PIs) enhance recombinant protein (RP) accumulation. (a) Four candidate PI selection strategies. (b) Details of the selected candidate PIs. *, presumed class of target proteases. References: [1] Luckett *et al*., 1999; [2] Arkadash *et al*., 2017; [3] Fluhr *et al*., 2012; [4] Kim *et al*., 2008; [5] Martinez *et al*., 2009; [6] Goulet *et al*., 2012; [7] Shindo *et al*., 2016; [8] Tian *et al*., 2004; [9] Tian *et al*., 2007; [10] Lozano‐Torres *et al*., 2012; [11] Kim *et al*., 2015. (c) Expression cassette for PIs containing a 35S promoter and terminator, NtPR1 signal peptide (SP) and PIV2 intron, flanked by T‐DNA borders. (d) PIs were transiently co‐expressed in *N. benthamiana* with three RPs [α‐Galactosidase (αGal), erythropoietin (EPO), and antibody VRC01] by agroinfiltration. (e) Screening results. Four PIs enhanced RP accumulation (black boxes) and 25 PIs had minor or no effect on RP accumulation (grey boxes). Effects of six PI‐RP combinations were not determined (ND).

To overcome the degradation bottleneck in molecular farming, we aimed to co‐express secreted recombinant proteins (RPs) with secreted protease inhibitors (PIs). We took four approaches to select candidate PIs (Figure 1a). First, we mined the literature for ten strong and/or stable inhibitors targeting each class of proteases, preferably selecting *N. benthamiana* proteins to simplify expression. We also included the dominant ubiquitin‐K48R mutant to block proteasome‐mediated degradation (Chau *et al*., 1989) and included native ubiquitin as a control. Second, we used public microarray data from the NCBI GEO database to select five putative plant PIs whose corresponding transcripts are depleted upon interaction with *A. tumefaciens* (Barrett *et al*., 2013; Ditt *et al*., 2006; Lang *et al*., 2016; Lee *et al*., 2009). Depleted putative inhibitors were chosen to shift the balance between active and inhibited proteases in agroinfiltrated leaves upon PI overexpression. In our third approach, we selected eight pathogen‐derived, secreted inhibitors known to target secreted plant immune proteases. Fourth, we identified four endogenous PIs associated with plant immunity, reasoning that they might both inhibit proteases and enhance plant fitness during interaction with *A. tumefaciens*. This resulted in a collection of 29 PIs (Figure 1b).

The 29 candidate PIs were cloned into a golden gate compatible binary vector containing a T‐DNA with a 35S promoter and terminator and an intron to exclude bacterial expression (Figure 1c, Vancanneyt *et al*., 1990; Engler *et al*., 2014). All constructs also carry the signal peptide (SP) of PR1a from *N. tabacum*, replacing the endogenous signal peptide, if present (Ohshima *et al*., 1987). To screen for PIs that enhance accumulation of a broad range of RPs, three model RPs were transiently co‐expressed with each candidate PI by agroinfiltration (Figure 1d). As model RPs, we used an α‐Galactosidase (αGal) used to treat Fabry's disease (Garman and Garboczi, 2002), the hematopoietic glycohormone erythropoietin (EPO) (Hayat *et al*., 2008) and the HIV‐neutralizing antibody VRC01 (Wu *et al*., 2010). During the screen, leaves were sampled at 3 days postinfiltration (3 dpi) and 6 dpi, or at 4 dpi and 7 dpi when P19 was co‐expressed. Each RP‐PI combination was tested at least once and PIs that enhanced RP accumulation in initial experiments were tested further, focusing on 3 dpi without P19 as the most informative condition (Figure 1e).

For benchmark purposes, the screen included three PIs that had been used previously to increase RP accumulation upon co‐expression, namely SlCYS8 (Jutras *et al*., 2016), SlCDI (Goulet *et al*., 2012) and the *N. benthamiana* protein most similar to *N. alata* protease inhibitor II (NbPot2, 87% identity) (Kim *et al*., 2008). However, SlCDI failed to increase RP accumulation in our system, possibly due to the differences in tested RPs and plant growth conditions. NbPot2 was also ineffective, possibly due to differences in RPs, expression systems and PI sequence. In contrast, SlCYS8 increased RP accumulation (Figure 1e), and SlCYS8 was thus used in further experiments as a positive control. The SlCYS8‐Q47P mutant PI lacks inhibitory activity (Sainsbury *et al*., 2013) and does not increase RP levels (Jutras *et al*., 2016) and was therefore included as a negative control.

### NbPR4, NbPot1 and HsTIMP enhance accumulation of three model RPs upon co‐expression

Of the 26 previously untested PIs, we identified three PIs that enhance RP accumulation upon co‐expression (Figure 1e). Besides PIs, we tested the dominant‐negative ubiquitin‐K48R mutant (Chau *et al*., 1989), but Ubi‐K48R did not change RP accumulation (Figure 1e). Interestingly, the three new PIs are unrelated and presumably target different protease classes. NbPR4 is the *N. benthamiana* protein most similar to CaPR4c (86.7% identical amino acids), a novel Cys protease inhibitor associated with defence against *Xanthomonas* in pepper (Kim and Hwang, 2015). NbPot1 (*N. benthamiana* Potato inhibitor type I of family I13) was initially selected for co‐expression because a transcript corresponding to a similar Arabidopsis protein was depleted upon interaction with *A. tumefaciens*. Indeed, also the level of the *N. benthamiana* transcript encoding NbPot1 (Niben101Scf00750XLOC_013210) is reduced 6.7‐fold at 2 days post agroinfiltration (Grosse‐Holz *et al*., 2018). HsTIMP was included in the candidate PI screen to target metalloproteases, for which we did not identify plant‐derived alternatives. HsTIMP is a well‐studied potential anticancer inhibitor targeting matrix‐metalloproteases (MMPs, family M10) (Arkadash *et al*., 2017). MMPs are also conserved in plants (Marino and Funk, 2012) and have been implicated on recombinant protein processing (Mandal *et al*., 2010). We also found that both transcripts and extracellular peptides corresponding to MMPs accumulate in agroinfiltrated leaves of *N. benthamiana* (Grosse‐Holz *et al*., 2018). The MMP inhibitory function of HsTIMP can be disrupted by appending an Ala residue to the N‐terminus of HsTIMP (Wingfield *et al*., 1999).

**Figure 2:**
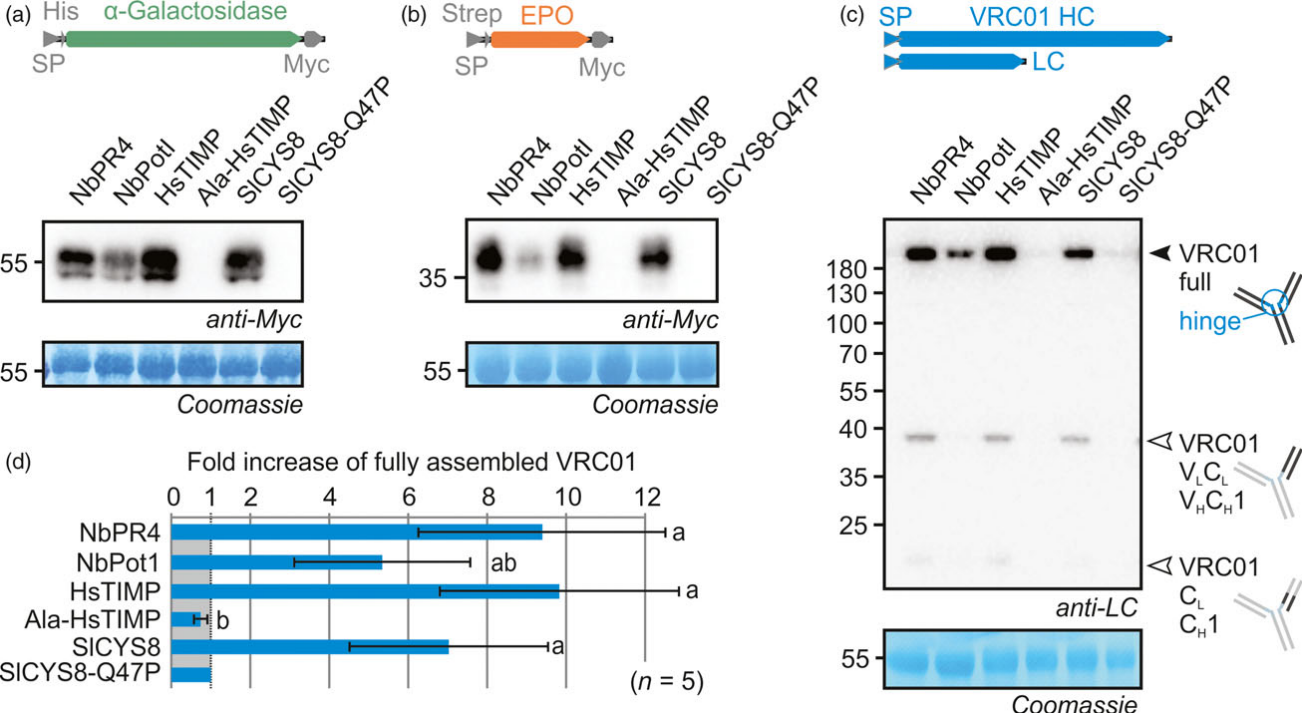
Individual co‐expression with NbPR4, NbPot1 or HsTIMP enhances accumulation of αGal (a), EPO (b) and VRC01 (c). Leaves were infiltrated with 1/1 (v/v) mixes of *A. tumefaciens* strains carrying plasmids for expression of αGal (a) or EPO (b) and PI or 1/1/1 (v/v) mixes of *A. tumefaciens* strains carrying plasmids for expression of VRC01 heavy chain, VRC01 light chain and PI (c). Full leaf extracts were harvested at 3 dpi. Proteins were subjected to reducing (a–b) or nonreducing (c) SDS‐PAGE and transferred onto PVDF membranes. RP accumulation was visualized using the indicated antibodies. Closed and open triangles in (c) indicate the full‐length VRC01 and putative degradation products, respectively (V_L_ /C_L_ , variable/constant domain of the light chain, V_H_ /C_H_ , variable/constant domain of the heavy chain). The blots are representative of at least five biological replicates. (d) The top band in VRC01 blots was quantified using ImageJ and normalized to the SlCYS8‐Q47P control (± SD, n = 5, ANOVA *P* < 0.0001 with *post hoc* Tukey test, *P* < 0.05). The 55 kDa Rubisco protein stained by Coomassie is shown as a loading control.

Co‐expression with NbPR4, NbPot1, HsTIMP or SlCYS8 enhanced accumulation of αGal when compared to the SlCYS8‐Q47P negative control (Figure 2a). Full‐length αGal without SP has a predicted molecular weight (MW) of 50.2 kDa and contains four *N‐*glycosylation sites, indicating that the band at ~55 kDa represents the intact mature glycoprotein. Importantly, co‐expression with Ala‐HsTIMP does not enhance αGal accumulation (Figure 2a), suggesting that inhibitory activity of HsTIMP is required to enhance RP accumulation. Interestingly, similar to αGal levels, accumulation of EPO is enhanced in the same way upon PI co‐expression (Figure 2b). This is remarkable because the PIs are unrelated and the RPs share no similarities. EPO without SP has a predicted MW of 23.4 kDa and contains three *N‐*glycosylation sites and one *O*‐glycosylation site, indicating that the band at ~35 kDa represents the intact mature glycoprotein.

The VRC01 antibody was transiently produced by co‐expressing the heavy and light chains (HC and LC) and detected using an anti‐κ LC antibody upon separation on nonreducing gels. Fully assembled VRC01 is detected at >180 kDa, similar to purified VRC01, indicating that PI co‐expression enhances accumulation of the fully assembled antibody, consisting of two HCs and two LCs (Figure 2c). Besides full‐length VRC01, we also detect VRC01 fragments which likely result from cleavages close to the hinge region and between the variable and constant domains of HC and LC. Cleavage at these positions is common for immunoglobulin G (IgG) type mAbs, and similar LC fragments were detected for other IgG mAbs (Donini *et al*., 2015; Hehle *et al*., 2015; Niemer *et al*., 2014).

Although the levels of all RPs are boosted upon PI co‐expression, the strength of this effect differs between RPs. Full‐length VRC01 accumulation is fivefold to 10‐fold enhanced, a change that lies within the dynamic range of Western blots and could thus be quantified directly (ANOVA *P* < 0.0001, *post hoc P* < 0.05) (Figure 2d). αGal and EPO, however, were not detectable in the control sample without overexposing the bands in PI co‐expressing samples. We therefore performed Western blots on dilution series of these samples. These experiments indicate that αGal accumulation is enhanced ~14‐, ~8‐, ~7‐ and ~4‐fold upon co‐expression with HsTIMP, NbPR4, SlCYS8 and NbPot1, respectively (Figure S1). Accumulation of EPO is enhanced even further, namely ~27‐, ~23‐, ~16‐ and ~18‐fold upon co‐expression with HsTIMP, NbPR4, SlCYS8 and NbPot1, respectively (Figure S1). For fully assembled VRC01, the values are within the range of those obtained by direct band quantification, namely ~8‐, ~10‐, ~10‐ and ~2‐fold increased accumulation upon co‐expression with HsTIMP, NbPR4, SlCYS8 and NbPot1, respectively (Figure S1). In summary, we discovered three PIs that enhance accumulation of three unrelated RPs upon co‐expression. NbPR4 and HsTIMP are superior when compared to NbPot1 and SlCYS8 in boosting RP accumulation.

### PIs accumulate in *N. benthamiana* leaves upon transient overexpression

**Figure 3:**
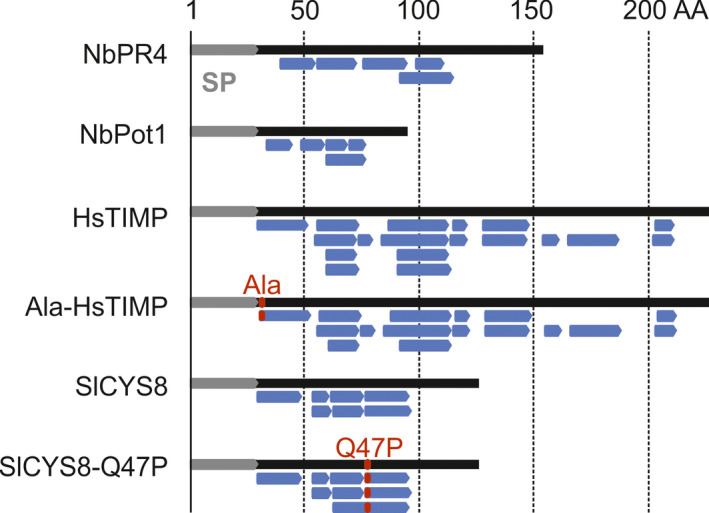
PIs accumulate in *N. benthamiana* leaves upon transient expression. Inhibitor‐derived peptides detected by MS in extracts obtained at 4 dpi from agroinfiltrated leaves co‐expressing PIs with P19. Peptides (blue) are mapped to the inhibitor sequences (black) which carry the NtPR1 signal peptide (SP, grey). For HsTIMP and SlCYS8, mutations are indicated in red.

To verify that both wild‐type and mutant PIs accumulate in leaves upon transient overexpression, we performed label‐free, quantitative mass spectrometry (MS) on extracts obtained at 4 dpi from agroinfiltrated leaves overexpressing the PIs in the presence of P19. Unique peptides derived from HsTIMP and SlCYS8 were identified in the respective extracts from leaves overexpressing both wild‐type and mutant PIs. Furthermore, peptides were identified that distinguished mutant from wild‐type PIs (Figure 3). Intensities of MS spectra corresponding to endogenous NbPot1‐derived peptides were below the detection threshold in all but the NbPot1‐overexpressing samples. In contrast, peptides derived from endogenous NbPR4 were detected in all samples, but NbPR4 abundance increased on average 69‐fold in the overexpressing samples when compared to P19 controls. All overexpressed PIs were among the top 25% of most abundant proteins in their respective samples (Table S1).

### Unrelated PIs additively enhance RP accumulation

**Figure 4:**
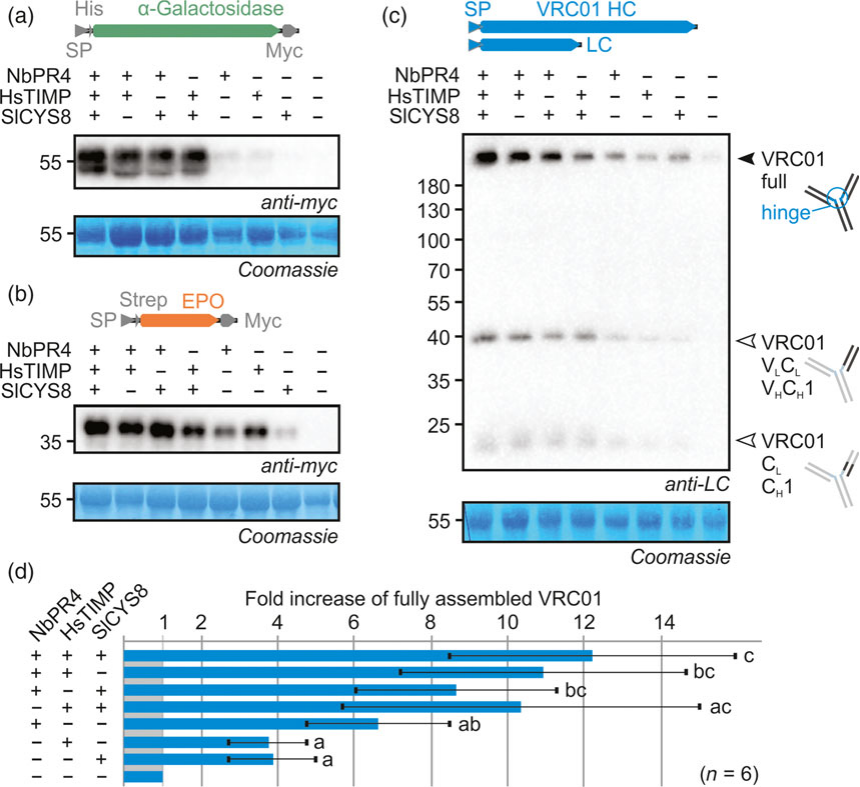
Combinations of NbPR4, HsTIMP and SlCYS8 enhance RP accumulation. Leaves were infiltrated with 1/1 (v/v) mixes of *A. tumefaciens* strains carrying plasmids for expression of αGal (a) or EPO (b) and PI or 1/1/1 (v/v) mixes of *A. tumefaciens* strains carrying plasmids for expression of VRC01 heavy chain, VRC01 light chain and PI (c). The PI part of the mixture contained three volumes of *A. tumefaciens* strains for expression of the indicated PIs, with one part mutant PI (Ala‐HsTIMP) used to replace each missing PI. Full leaf extracts were harvested at 3 dpi. Proteins were subjected to reducing (a–b) or nonreducing (c) SDS‐PAGE and transferred onto PVDF membranes. RP accumulation was visualized using the indicated antibodies. The blots are representative of six biological replicates each. (d) The top band in all VRC01 blots was quantified using ImageJ and normalized to the control (± SD, *n* = 6, ANOVA *P* < 0.0001 with *post hoc* Tukey test, *P* < 0.05). The 55 kDa Rubisco protein stained by Coomassie is shown as a loading control.

As NbPR4, NbPot1, HsTIMP and SlCYS8 (PI families I43, I13, I35 and I25, respectively) are predicted to target different protease classes (Cys, Ser, metallo‐ and Cys proteases, respectively), we tested whether they could act together to enhance RP accumulation. From an initial screen of all binary combinations between the four, we found that combinations without NbPot1 increased RP accumulation the most (Figure S2). The triple combination consisting of NbPR4, HsTIMP and SlCYS8 further enhanced accumulation of αGal, EPO and VRC01 (Figure 4a–c). Quantification of full‐length VRC01 signals showed significantly higher VRC01 accumulation upon co‐expression with the triple PI combination when compared to co‐expression with a single PI in combination with a mutant PI (ANOVA *P* < 0.0001, *post hoc P* < 0.05) (Figure 4d). However, RP levels were similarly increased when threefold more bacteria with PI‐encoding T‐DNA were infiltrated (Figure S3). Therefore, the additive effects seem to be caused by increased PI levels and not by synergy between PIs, indicating the effect of each PI is dose‐dependent.

### NbPR4, NbPot1 and HsTIMP differ from SlCYS8 in their effect on protease activity profiles

**Figure 5:**
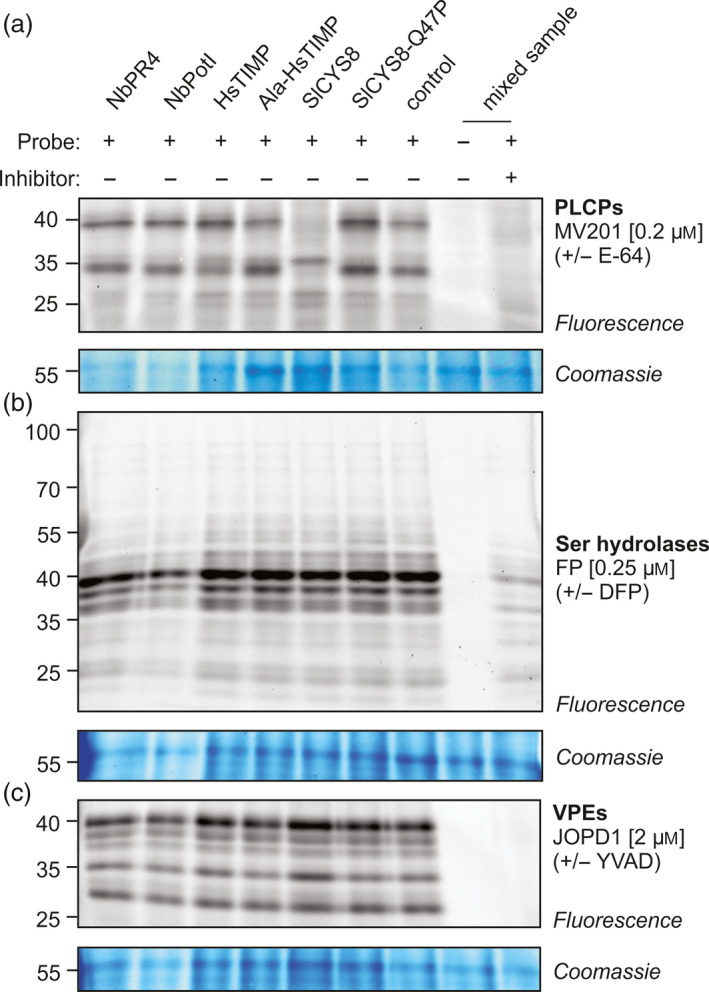
Activity‐based protein profiling of cellular proteases indicates that NbPR4, NbPot1 and HsTIMP differ from SlCYS8 in their mode of action. Activity profiles of papain‐like Cys proteases (PLCPs, (a), Ser hydrolases (SHs, (b) and vacuolar processing enzymes (VPEs, (c) Leaves were infiltrated with *A. tumefaciens* harbouring the indicated PI expression plasmid, mixed 1/1 (v/v) with *A. tumefaciens* harbouring the P19 expression plasmid. Leaf extracts (pH 5) were obtained at 4 dpi, adjusted to the same protein concentration, and 48 μL of each sample was pre‐incubated with or without 0.2 mm of inhibitor (E‐64, DFP or YVAD) for 30 min and then incubated with or without the indicated probe for 4 h (MV201, JOPD1) or 1 h (FP) at room temperature. Labelled proteins were visualized by in‐gel fluorescence scanning. The 55 kDa Rubisco protein stained by Coomassie is shown as a loading control.

To understand how NbPR4, NbPot1 and HsTIMP enhance RP accumulation, we investigated how these PIs affect activity of *N. benthamiana* proteases in agroinfiltrated leaves. We thus characterized extracts harvested at 4 dpi from leaves transiently overexpressing each PI. We assessed activity of specific protease families using activity‐based protein profiling (ABPP), which is based on the labelling of proteomes with fluorescent chemical probes that react covalently with the active site of enzymes in an activity‐dependent manner (Morimoto and van der Hoorn, 2016). We surveyed active papain‐like Cys proteases (PLCPs, family C01), Ser hydrolases (SHs) and vacuolar processing enzymes (VPEs, family C13) using the activity‐based probes MV201 (Richau *et al*., 2012), FP‐TAMRA (Kaschani *et al*., 2009) and JOPD1 (Lu *et al*., 2015), respectively. SlCYS8, but not SlCYS8‐Q47P, suppresses PLCP activity (Figure 5a), consistent with an independent study (P. Varennes‐Jutras, F. Grosse‐Holz & R. van der Hoorn, unpublished data). In contrast to SlCYS8, overexpression of NbPR4, NbPot1, HsTIMP or Ala‐HsTIMP does not affect PLCP activity when compared to the control (Figure 5a). This is remarkable because we expected that NbPR4 inhibits Cys proteases, like CaPR4c in pepper (Kim and Hwang, 2015). Activity profiles of both SHs and VPEs are unaffected by PI overexpression, even though NbPot1 is annotated as a family I13 Ser protease inhibitor. To test specifically whether extracellular proteases were affected by overexpression of the PIs, we also surveyed extracellular PLCPs, SHs and VPEs using ABPP in apoplastic fluid isolated from PI‐overexpressing leaves. Also there, SlCYS8 overexpression affects activity of extracellular PLCPs when compared to SlCYS8‐Q47P overexpression or to the control (Figure S6, Jutras *et al*., manuscript in preparation). Expression of NbPot1, HsTIMP and Ala‐HsTIMP did also not affect the activity of extracellular PLCPs, SHs and VPEs (Figure S4).

### NbPR4, NbPot1 and HsTIMP similarly affect leaf proteomes

We hypothesized that PI overexpression might affect the accumulation of endogenous proteins. For instance, protease substrates might accumulate, inhibited proteases might be degraded, or PIs might be sensed by the plant and trigger a response. We thus also analysed endogenous differentials in the proteomes from leaves overexpressing the PIs, exploring the same proteomics data set used for Figure 3 (Table S1).

Besides the overexpressed PIs and P19, we quantified peptides corresponding to 3349 endogenous *N. benthamiana* and *A. tumefaciens* proteins. Of these, 3236 (97%) remained unchanged upon overexpression of any PI compared to the P19 control, indicating that the PIs have rather subtle effects on the leaf proteome. However, 167 (3.4%) proteins changed significantly (Student's *t*‐test, *P* < 0.05) and more than twofold in abundance when comparing leaves overexpressing each PI separately to the control (P19 only) (Table S1).

We detected 219 proteases in this shot‐gun proteomics experiment (Table S1). These proteases represent 41 families and include 17 Asp, 45 Cys, 85 Ser, 31 Thr and 41 metalloproteases. However, the predicted target proteases of the PIs (Cys proteases for NbPR4 and SlCYS8, Ser proteases for NbPot1 and metalloproteases for HsTIMP) did not change significantly and more than twofold in abundance when comparing proteomes of PI‐overexpressing leaves to their respective controls.

**Figure 6:**
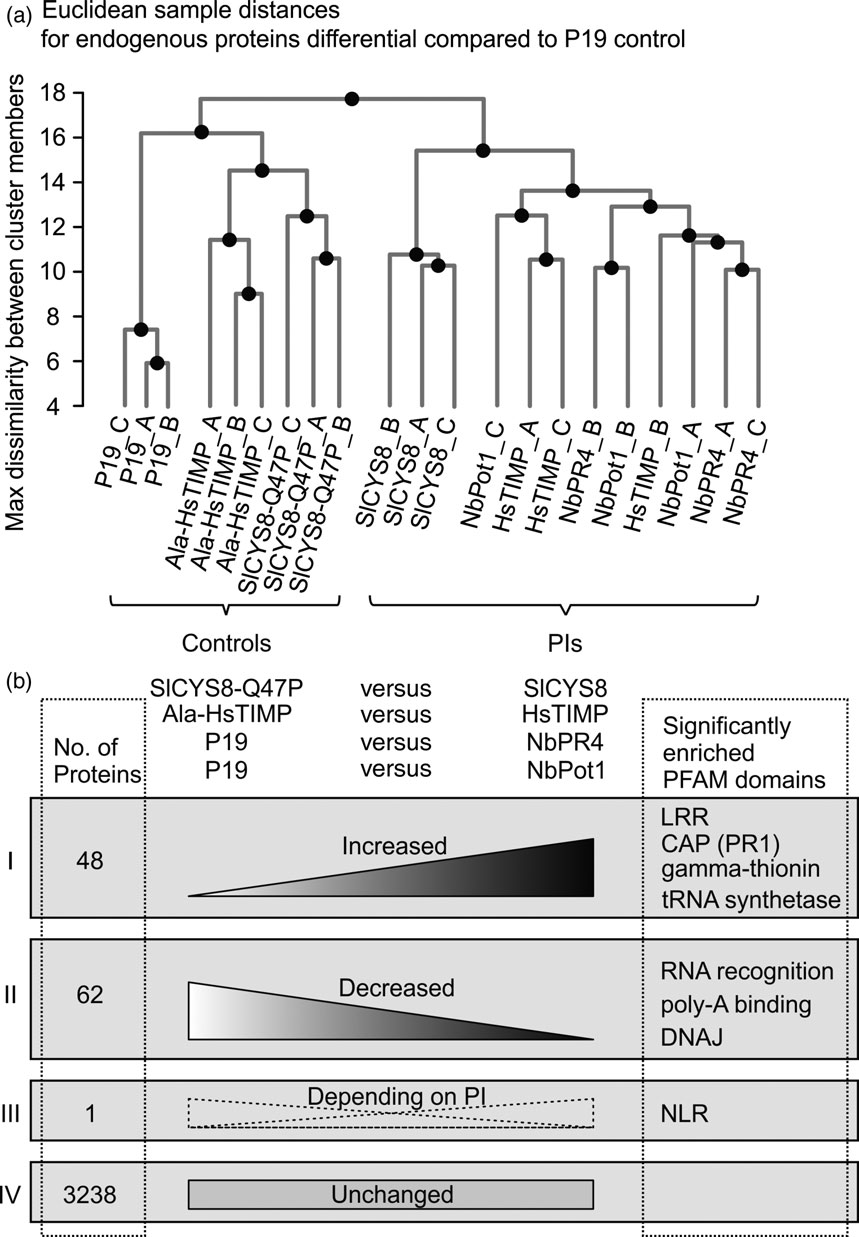
NbPR4, NbPot1 and HsTIMP similarly affect leaf proteomes. Label‐free quantitative MS was performed on leaf extracts (4 dpi) from leaves overexpressing the PIs in the presence of P19. (a) Complete linkage clustering was performed using Euclidean distances between samples, based on all endogenous differential proteins. Endogenous differential proteins are all proteins that differ significantly (*t*‐test, *P* < 0.05) and >2‐fold in abundance between PI‐overexpressing and P19‐expressing control leaves, but not P19 and PIs. (b) Differentials associated with PI overexpression are endogenous proteins that differ significantly (*t*‐test, *P* < 0.05) and >2‐fold when comparing NbPR4 and NbPot1 expressing leaves to the P19 control and HsTIMP and SlCYS8 expressing leaves to the respective mutant PI control. Proteins were grouped according to whether they increased for at least one PI and never decreased (group I), decreased for at least one PI and never increased (group II), increased for some, but decreased for other PIs (group III) or never changed significantly and more than twofold (group IV). PFAM domains named with groups I to III are enriched (geometric test, Benjamini‐Hochberg adjusted *P* < 0.05) among the proteins in the respective group, representative PFAMs are shown.

Notably, Euclidean distance clustering of only these endogenous differential proteins reveals two subclusters: The samples from leaves overexpressing the PIs form subcluster A and the controls form subcluster B (Ala‐HsTIMP, SlCYS8 Q47P and P19) (Figure 6a). Interestingly, the samples from leaves overexpressing HsTIMP, NbPot1 or NbPR4 cluster together and separately from SlCYS8 overexpressing leaf samples within subcluster A. The effects of both SlCYS8 and HsTIMP on the endogenous proteome thus depend on protease inhibitory activity, and the effect of SlCYS8 is distinct from the effects of HsTIMP, NbPot1 and NbPR4, which have very similar effects on the leaf proteome.

We next analysed the endogenous differentials that distinguish the effect of SlCYS8 from the effect of other PIs, but their annotations are associated only with general cellular processes and thus rather inconclusive (Table S1). More targeted approaches such as activity‐based proteomics or the identification of SlCYS8 interactors may be required to resolve how the impact of SlCYS8 differs from the other PIs.

To elucidate what distinguishes the PI‐overexpressing leaf samples in subcluster A from the controls in subcluster B, we determined which proteins changed significantly (Student's *t*‐test, *P* < 0.05) and more than twofold in abundance when comparing NbPR4 and NbPot1 overexpressing leaves to the P19 control and SlCYS8 and HsTIMP overexpressing leaves to their respective mutant PI controls (Figure 6b). We divided the differential proteins into three groups: increased in abundance upon overexpression of at least one PI compared to the respective control and not decreased for another PI (*n* = 48 proteins, group I); decreased upon overexpression of at least one PI compared to the respective control and not increased for another PI (*n* = 62 proteins, group II); and increased for one/some PI(s), but decreased for other(s) (*n* = 1 protein, group III). Importantly, the PIs have very similar effects on the leaf proteome, as only one protein was differential in opposing directions (group III). This one protein is a nucleotide‐binding, leucine‐rich repeat immune receptor (NLR, Niben101Scf02118g00018), which decreased in abundance upon HsTIMP overexpression when compared to Ala‐HsTIMP overexpression, but increased in abundance for all other PI/control comparisons.

We next analysed the PFAM family annotation of the proteins in groups I and II as an indicator for protein function. PFAM families associated with transcription and translation are enriched among both increasing and decreasing proteins (hypergeometric test, Benjamini‐Hochberg adjusted *P* < 0.01) (Figure 6b and Table S1). This includes tRNA synthetases (two proteins) among the proteins increasing in abundance and families of RNA recognition (three proteins), poly‐A binding (two proteins) and chaperone DNAJ (one protein) among the proteins decreasing in abundance upon PI overexpression. Most of these proteins have several homologues within *N. benthamiana*, which might replace each other. For instance, we detect 59 proteins carrying an RNA recognition motif (PF00076) domain in leaves, five of which are differential in either direction for at least one PI versus control comparison.

Interestingly, domains associated with plant defence are also enriched among the proteins increasing in abundance upon PI overexpression. This includes leucine‐rich repeat (LRR) domains that are part of plant immune receptors (three proteins), cysteine‐rich secretory protein (PR1, one protein) and gamma thionin (one protein). Gamma thionins are small, stable plant defense proteins, many of which have antifungal properties or act as protease inhibitors (Pelegrini and Franco, 2005).

We also identified peptides corresponding to 68 proteins from Agrobacterium, four of which changed in abundance upon PI expression (Table S1). Further studies will be needed to confirm differential accumulation of the detected endogenous proteins.

## Discussion

We tested 29 candidate PIs for enhancing accumulation of three RPs upon co‐expression by agroinfiltration. Of the 29 tested candidate PIs, four enhance RP accumulation upon co‐expression. The remaining 25 can be regarded negative controls. Interestingly, we tested six cystatins (family I25), but only SlCYS8 enhances RP accumulation. Similarly, we tested three PIs of family I13, but only NbPot1 enhances RP accumulation. Related proteins that do not enhance RP accumulation can be used in domain swap and mutagenesis experiments to determine essential sequence and/or structural motifs. In combination with ABPP, this approach could reveal which proteases or protease subfamilies are inhibited to enhance RP accumulation.

### Unrelated protease inhibitors affect unrelated recombinant proteins in similar ways

The model RPs used in our screen were the lysosomal enzyme α‐Galactosidase (αGal), the small glycohormone erythropoietin (EPO) and VRC01, an immunoglobulin G (IgG) type antibody. We chose completely unrelated model RPs (<23% identity between any two), reasoning they were likely to differ in sensitivity to proteases and to be degraded by different proteolytic pathways. Surprisingly, RP accumulation is affected in the same way for αGal, EPO and VRC01. HsTIMP and NbPR4 enhance accumulation more than NbPot1, which has a similar effect to SlCYS8. Our data indicate that the degradation processes of unrelated RPs must have something in common that can be affected by PIs to produce the same outcome for each RP.

Astonishingly, the four unrelated PIs (<13% identical amino acids between any two) have similar effects although they probably target different proteases. HsTIMP presumably inhibits matrix‐metalloproteases and NbPot1 belongs to the I13 family, which targets Ser proteases (Rawlings, 2016; Wingfield *et al*., 1999). SlCYS8 inhibits PLCPs (Figure 5a, Jutras *et al*., manuscript in preparation). NbPR4 likely also targets Cys proteases, as it is 86.7% identical to the pepper Cys protease inhibitor CaPR4c (Kim and Hwang, 2015). However, activities of PLCPs that are inhibited by SlCYS8 are not affected by NbPR4, indicating NbPR4 either targets different Cys proteases or acts in a different way.

### Three possible proteolytic mechanisms

**Figure 7:**
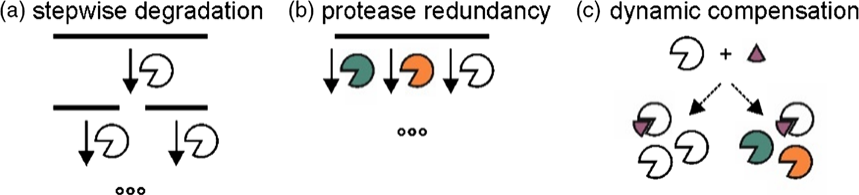
Models for RP degradation mechanisms.

Different proteolytic mechanisms of RP degradation can explain how three unrelated RPs are affected similarly by three unrelated PIs. The first proteolytic mechanism is that degradation happens stepwise (Figure 7a). An initial cleavage exposes parts of the protein that are then susceptible to many different proteases. Downstream degradation can thus be hindered by unrelated PIs. This may be the case for VRC01, where we detect fragments indicating that an initial cleavage happens in the hinge region, followed by downstream degradation of the fragments. None of the PIs cause a shift in the relative abundances of the VRC01 fragments, indicating that the initial cleavage is not blocked by any of the four PIs. In accordance with our results, N‐terminal sequencing of antibody fragments revealed that SlCYS8 co‐expression does not prevent initial cleavage of the human IgG H10 (Jutras *et al*., 2016). We do not detect fragments of αGal and EPO, indicating that fragments of αGal and EPO do either not accumulate or lack the myc tag epitope used for detection.

The second proteolytic mechanism is that different proteases act redundantly in RP degradation (Figure 7b). PIs can thus act on different proteases to decrease the pool of total proteolytic activity. This may be the case, as accumulation of the same RP can be enhanced by different PIs. In this scenario, one would expect different PIs to have additive effects when combined. Indeed, we detected increased RP accumulation when co‐expressing two or three PIs compared to one PI and a mutant PI. However, we also observed that the effect of each single PI on RP levels is dose‐dependent. RPs accumulate more when one‐third of the agroinfiltration mixture delivers PI‐encoding T‐DNA than when only one of nine of the mixture delivers PI‐encoding T‐DNA.

At the concentrations we used, we expect Agrobacteria to transform virtually all cells in the infiltrated zone (Buyel *et al*., 2015; Castilho *et al*., 2014). Consequently, expression driven by a 35S promoter should cause accumulation of PIs in nearly every cell. Two mechanisms can explain why we still observe a concentration‐dependent effect of PIs on RP levels. First, genes on extrachromosomal T‐DNA are expressed *in planta* (Mysore *et al*., 2000; Singer *et al*., 2012). Delivery of more T‐DNAs may therefore increase PI expression further, even if all cells in the infiltrated area are transformed already. Second, the transcription/translation machinery in agroinfiltrated leaves may be running at full capacity, and co‐expression of additional proteins may therefore divert resources away from RP and PI expression.

The third proteolytic mechanism is that protease activity is dynamic, so that inhibition can be compensated (Figure 7c). Plants may be able to sense the lack of protein turnover upon protease inhibition and trigger the up‐regulation of proteases to ensure homoeostasis. Increased transcription of protease‐encoding genes upon protease inhibition is seen for instance for the proteasome, for which encoding genes show increased transcript levels in the presence of the proteasome inhibitor syringolin A (Michel *et al*., 2006). Protein levels of proteasome subunits remain constant, indicating that transcriptional up‐regulation compensates for degradation of inhibited subunits (Svozil *et al*., 2014). We found that the abundance of predicted target proteases does not increase in proteomes of PI‐overexpressing leaves. Transcriptomes of PI‐overexpressing leaves could be analysed to test whether protease transcription increases upon PI overexpression. Protease activity may also be compensated by post‐translational activation of a nontarget protease, which then compensates for the inhibited PI targets. The proteases we monitored here (PLCPs, SHs and VPEs) do not show increased activity‐based labelling, but the compensatory proteases could also be aspartic and metalloproteases or other proteases that are not displayed under the tested conditions. As a broader approach, N‐terminal proteomics (e.g. TAILS, COFRADIC) could reveal neo‐N‐termini of activated proteases, whereas gel‐based proteomics (PROTOMAP) could be used to detect shifts in the size of proteases upon release of inhibitory prodomains (Dix *et al*., 2008; Huesgen and Overall, 2012).

### Protease inhibitor overexpression may trigger plant immunity

The three new PIs that enhance RP accumulation have small and remarkably similar effects on the leaf proteome when compared to mutant PIs and controls. PFAM families enriched among the proteins that change in abundance upon PI overexpression is associated with increased abundance of immune signalling components. Protease inhibition thus seems to not only prevent RP degradation, but also trigger some kind of immune response. T‐DNA delivery is limited by immune responses in flowering *N. benthamiana* triggered by the perception of cold shock protein of Agrobacterium (Saur *et al*., 2016), highlighting the impact of plant immunity on the performance of the agroinfiltration expression platform. Investigating how the interaction between *N. benthamiana* and *A. tumefaciens* is altered by PI overexpression and whether this also affects T‐DNA delivery or RP expression rates will be an interesting topic for further studies.

### PIs are tools to unravel the proteolytic network degrading RPs

As RP degradation in agroinfiltrated leaves is at least partially independent of RP sequence and structure, unravelling the underlying proteolytic network will be highly valuable. PIs can be instrumental to understand degradation or processing when proteases act redundantly. For instance, the peptide hormone IDA (Inflorescence Deficient in Abscission) is processed by several subtilases that act redundantly (Schardon *et al*., 2016). A role for subtilases was found upon overexpression of the Kazal‐like Ser protease inhibitor EPI10 from *Phytophthora infestans*, which targets subtilases (Tian *et al*., 2004) and prevents abscission in Arabidopsis (Schardon *et al*., 2016). Abscission can be restored in EPI10 overexpressing plants by supplying mature IDA peptide and indeed, multiple target subtilases of EPI10 can process IDA *in vitro* (Schardon *et al*., 2016). Analogously, target proteases of NbPot1, NbPR4 and HsTIMP are implicated in RP degradation. For PIs that do not deplete protease activities that we can detect in ABPP, immunoprecipitation of PIs from leaf extracts may reveal co‐purifying target proteases. We have performed preliminary experiments with N‐ and C‐terminal tags, but these disrupted the RP accumulation enhancing function of the PIs. NbPR4, NbPot1 and HsTIMP thus likely require intact termini for inhibitory activity. Specific antibodies raised against the plant‐produced PIs or immobilization of purified PIs may facilitate future immunoprecipitation experiments to identify the targets of these PIs.

## Conclusions

We have identified three new, unrelated PIs that enhance accumulation of three unrelated RPs to a similar extent. Our data indicate that RPs are degraded by a dynamic protease network, where different proteases act redundantly and/or compensatory. NbPR4, NbPot1 and HsTIMP can be used to unravel the *N. benthamiana* protease network. Importantly, PI co‐expression substantially increases accumulation of three unrelated RPs, suggesting that NbPR4, NbPot1 and HsTIMP may increase the levels of a broad variety of RPs in industrial molecular farming. We thus discovered novel tools to improve the general productivity of the agroinfiltration expression platform.

## Experimental procedures

All chemicals and oligonucleotides were obtained from Sigma (Sigma‐Aldrich, St. Louis, MO) unless specified otherwise.

### Cloning of PIs and RPs

The Golden Gate Modular Cloning (MoClo) kit (Weber *et al*., 1990) and the MoClo plant parts kit (Engler *et al*., 2014) was used for cloning, and all vectors are from this kit unless specified otherwise. Generated and used plasmids and primers are summarized in Tables S2 and S3, respectively. The binary vector for expression, pJK001, was created by combining pAGM4723, pICH47732, and pICH41722 in a BpiI reaction, creating a level 2 plasmid based on pAGM4723 in which one transcriptional unit can be cloned using BsaI. pJK002 (Figure 1c) was constructed by taking the sequence encoding the first 30 amino acid residues of *Nicotiana tabacum* PR1a (GenBank ×06361), constituting its N‐terminal signal peptide, with a synonymous substitution of C>A in the last nucleotide of the last codon. The Portable intron (PIV2), derived from the second intron (IV2) of the potato gene ST‐LS1 (Vancanneyt *et al*., 1990), was inserted after the first nucleotide coding for the glycine‐glycine overhang in a consensus overhang for plant introns (AG//GTAAGT…TGCAG//G), ensuring no potential alternative in‐frame ATG start codons were present. This sequence was synthesised by Biomatik and cloned into pICH41258. The sequence encoding for the Tomato bushy stunt virus P19 silencing inhibitor (GenBank AJ288941; isolate To4B) was synthesized as a GeneArt String (ThermoFisher) including an additional ATG start codon. This sequence was cloned into pICH41308 creating pJK046. The P19 sequence from pJK046 was placed in between the double Cauliflower Mosaic Virus 35S promoter with the Tobacco Mosaic Virus omega 5′UTR (pICH51288) and the Cauliflower Mosaic Virus 35S 3′UTR and polyadenylation signal/terminator (pICH41414) in the binary vector pJK001 creating pJK050. The sequence encoding NbPR4 was amplified from *N. benthamiana* genomic DNA using primers #001 (including the native SP) or #005 (without the native SP) and #003 and cloned into pICH41308 (including the native SP) or pICH41264 (without the native SP), respectively. The sequence encoding HsTIMP was codon‐optimized for *in planta* expression (Table S4), synthesized as a GeneString (Thermo Fisher Inc, Waltham, MA, USA) and cloned into pICH41264 (without the native SP). The sequence encoding NbPot1 was amplified from *N. benthamiana* genomic DNA using primers #074 and #075 and cloned into pICH41264. Other inhibitors were cloned analogously; oligonucleotides designated as ‘s’ (sense) or ‘as’ (antisense), respectively, were annealed according to the manufacturer's instructions and added to the golden gate reaction, where primers are designated ‘p1’, ‘p2’ etc., inhibitors were cloned in multiple parts to domesticate the sequences. Each inhibitor level 0 module was then combined with pICH51288, pJK002, pICH41414 and pJK001 in a BsaI reaction to obtain the expression plasmids pFGH008 (NbPR4), pFGH053 (NbPot1) and pFGH047 (HsTIMP). All other PIs were cloned analogously, with the exceptions of SFTI1, which retained its native signal peptide as this may be required for correct processing and folding into the rigid structure that defines this PI (Luckett *et al*., 1999), and NbK1‐3, which were cloned into pL0V‐C1‐15457 (without SP, for N‐ and C‐terminal tagging (ENSA consortium https://www.ensa.ac.uk/)) and combined pFGH074 (generated from pL0M‐C2‐FLAG‐15198 (ENSA consortium https://www.ensa.ac.uk/) by adding 5′‐tacccatacgatgttccagattacgct‐3′) to clone them with a C‐terminal FLAG‐HA tag. The sequences of VRC01 heavy chain (HC) and light chain (LC), codon‐optimized for plant expression, were amplified from plasmids kindly provided by Julian Ma (St. George's, University of London) (Teh *et al*., 2014) and cloned into pICH41308 (including the native SP). Each level 0 module was then combined with pICH51288, pICH41414 and pJK001 in a BsaI reaction to obtain the expression plasmids pLM15 (VRC01 LC) and pLM16 (VRC01 HC). The sequences encoding EPO and αGal were codon‐optimized for in planta expression (Table S4), synthesized as GeneStrings (Thermo Fisher Inc) and cloned into pL0V‐C1‐15457. The αGal and EPO level 0 modules were combined with pICH45089, pLM07 (pL0M‐U‐TMV/NtPR1a, generated from pICH41402 by insertion of the NtPR1SP), pL0M‐S‐6xHis‐15258 (ENSA consortium https://www.ensa.ac.uk/) for αGal or pLM09 (pL0M‐S‐StrepII, generated by inserting 5′‐tggtcacatcctcaatttgaaaag‐3′ into pICH41258) for EPO, pL0M‐C2‐3xMyc‐15212 (ENSA consortium https://www.ensa.ac.uk/), pICH41414 and pJK001 in a BsaI reaction to obtain the expression plasmids pLM25 (EPO) and pLM34 (αGal).

Plasmids were transformed into *E. coli* for amplification, purified, sequenced and transformed into *Agrobacterium* GV3101‐pMP90. *Agrobacterium* GV3101‐pMP90 was cultured on plates of LB medium (10 g/L NaCl, 10 g/L Tryptone, 5 g/L yeast extract, 15 g/L agar) containing 25 μm rifampicin, 50 μm gentamycin and 50 μm kanamycin to select for transformants. A single colony was picked and cultured in liquid LB medium (10 g/L NaCl, 10 g/L Tryptone, 5 g/L yeast extract) containing 25 μm rifampicin, 50 μm gentamycin and 50 μm kanamycin. Glycerol stocks were prepared by mixing this culture 1/1 (v/v) with 50% glycerol in water, flash‐freezing in liquid nitrogen and storing at −80°C. For each agroinfiltration experiment, a 160 μL aliquot of glycerol stock was thawed and inoculated into fresh LB.

### Agroinfiltration

*Nicotiana benthamiana* plants were grown at 21°C under a 16/8 h light/dark regime in a growth room. *Agrobacterium* GV3101‐pMP90 (WT), *Agrobacterium* GV3101‐pMP90 carrying a P19‐encoding plasmid (pJK050) or *Agrobacterium* GV3101‐pMP90 carrying EPO, αGal, VRC01 or inhibitor encoding plasmids were grown for 21 h at 28°C with agitation in LB containing 25 μm rifampicin and 50 μm gentamycin (for WT) plus 50 μm kanamycin (for *A. tumefaciens* harbouring plasmids). Bacteria were collected by centrifugation at 2000 ***g*** for 5 min at room temperature (RT), resuspended in infiltration buffer (10 mm 2‐(N‐morpholino) ethanesulfone (MES), 10 mm MgCl_2_, pH 5.7, 100 μm acetosyringone) to OD_600 _= 0.5 (OD_600 _= 1 for αGal) and left for 2 h at 28°C with agitation to recover. For co‐expression experiments, *A. tumefaciens* suspensions were mixed in the appropriate ratios (described in Figure legends). The first and second fully expanded leaves of preflowering *N. benthamiana* (4–5 weeks old) were infiltrated with the bacteria suspension using a 10‐mL syringe without a needle. For comparison, different *A. tumefaciens* suspension mixes were infiltrated into different sectors of the same leaf.

### Protein extraction and Western blot

Leaf extracts were prepared at three days postinfiltration (dpi), unless specified otherwise. Four leaf discs (22 mg each) from four individual plants were combined per sample, flash‐frozen in liquid nitrogen and pulverized in a TissueLyser ball mill (Qiagen, Hilden, DE). The tissue powder was mixed with 3/1 (v/fresh weight) cold phosphate‐buffered saline (PBS, 10 mm PO_4_^3−^, 137 mm NaCl, 2.7 mm KCl) and centrifuged for 10 min at 16 000 ***g*** and 4°C. The supernatant was mixed with 4x gel loading buffer (200 mm Tris‐HCl (pH 6.8), 400 mm DTT, 8% SDS, 0.4% bromophenol blue, 40% glycerol), heated for 5 min at 95°C and separated on Bis‐Tris gels at 100 V. Proteins were then transferred to a PVDF membrane using the TransBlot Turbo system (Bio‐Rad, Hercules, US). The membrane was blocked in 5% milk in TBS (50 mm Tris‐Cl, pH 7.6; 150 mm NaCl) for 1 h at room temperature (RT), incubated in 1/5000 anti‐myc‐HRP (ab1326, Abcam, Cambridge, UK) for detection of αGal and EPO or in 1/2000 anti‐kappa‐HRP (Sigma A7164) or 1/2000 anti‐gamma‐HRP (Sigma A6029) for detection of VRC01 overnight at 4°C and washed in TBST (0.005% Tween‐20) prior to detection with Clarity ECL substrate (Bio‐Rad).

### Protein extraction for ABPP‐MS

Leaf extracts were prepared at four days postinfiltration (dpi), to allow for maximum protein accumulation in the presence of the p19 silencing suppressor. Infiltrated tissue from three or four individual plants was combined per sample, weighed, flash‐frozen in liquid nitrogen and pulverized using pestle and mortar. The tissue powder was mixed with 3/1 (v/fresh weight) cold 500 mm NaAc, pH 5, 500 mm DTT and centrifuged for 45 min at 3500 ***g*** and 4°C. Protein concentrations were determined using a linearized Bradford assay (Ernst and Zor, 2010).

### Apoplastic fluid (AF) extraction

Six *N. benthamiana* leaves per sample were detached and vacuum‐infiltrated with ice‐cold water, dried on the surface and placed in a syringe without needle and plunger that was inserted in a 50‐mL falcon tube. AF was collected by centrifugation at 2000 ***g***, 4°C for 25 min and used immediately.

### ABPP

Leaf extracts made in or apoplastic fluid adjusted to 500 mm NaAc, pH 5, 500 mm DTT were used for labelling. Samples of 48 μL were pre‐incubated with or without 0.2 mm of inhibitor (E‐64, DFP or YVAD) for 30 min and then incubated with or without the indicated probe for 4 h (0.2 μm MV201, 2 μm JOPD1) or 1 h (0.25 μm FP) at room temperature. FP was obtained from Thermo (88318, TAMRA‐FP), and MV201 and JOPD1 were synthesized as described (Lu *et al*., 2015; Richau *et al*., 2012). ABPP reactions were terminated by adding 1 mL cold acetone. Samples were centrifuged for 3 min at 16 000 ***g***, the supernatant discarded and the proteins resuspended in 2 x gel loading buffer (100 mm Tris‐HCl (pH 6.8), 200 mm DTT, 4% SDS, 0.02% bromophenol blue, 25% glycerol), heated for 5 min at 95°C and separated on Bis‐Tris gels at 100 V. Fluorescence scanning was performed on a Typhoon scanner (Amersham/GE Healthcare, Little Chalfont, UK), using Cy3 settings.

### Mass spectrometry

#### Sample preparation, reduction/alkylation and tryptic In‐Solution Digestion (ISD)

Full leaf extracts (FLE) were diluted to 0.5 mg/mL in 500 mm NaAc with 5 mm DTT (pH 5). An equivalent of 15 μg total protein (based on Bradford assay) was transferred into a fresh Eppendorf tube. Proteins were then precipitated by addition of 600 μL ice‐cold methanol, followed by 150 μL ice‐cold chloroform and 450 μL ice‐cold MS grade water. After 1 min centrifugation at 14 000 ***g*** and room temperature (RT), the upper aqueous phase was discarded, and 450 μL methanol was added. The precipitated proteins were collected by centrifugation (18 000 ***g***, 2 min), and supernatant was removed. The pellet was dried at room temperature (RT) for 5 min and then taken up in 49 μL 50 mm Tris‐HCl pH 8 containing 8 m Urea. Protein reduction and alkylation were achieved by sequential incubation with dithiothreitol (DTT, final 5 mm, 30 min, RT) and iodoacetamide (IAM; final 20 mm, 30 min, RT). To start protein digestion under denaturing conditions (urea concentration after reduction/alkylation is still 6.4 m), we added 400 ng trypsin/LysC mix (Promega). The samples were incubated at 37°C for 3 h while gently shaking (600 rpm). The samples were then diluted with 900 μL 50 mm Tris‐HCl pH 8 to a final urea concentration <1 m and incubated at 37°C overnight while gently shaking (600 rpm). The protein digestion was stopped by adding formic acid (FA, final 1% v/v). Acidified tryptic digests were desalted using Sep‐pak columns (Waters, 610 Centennial Park, Herts, UK). Columns were flushed with 5 mL elution buffer (65% acetonitrile, 0.1% TFA), and peptides were loaded onto column in 10 mL of washing buffer (2% acetonitrile, 0.1% TFA). Column was rinsed with 10 mL washing buffer, and peptides were eluted with 1.2 mL elution buffer. Samples were dried in a vacuum concentrator (Eppendorf).

#### Sample clean‐up for LC‐MS

Samples for MS were further desalted using self‐made C18 Stage Tips as described previously (Rappsilber *et al*., 2007). Briefly, dried peptides were dissolved in 100 μL 0.1% FA and loaded on a two‐disc StageTip by centrifugation (600–1200 ***g***). Bound peptides were washed with 0.1% FA and subsequently eluted with 80% Acetonitrile (ACN). After elution from the StageTips, samples were dried using a vacuum concentrator (Eppendorf), and the peptides were taken up in 10 μL 0.1% FA.

#### LC‐MS/MS

Experiments were performed on an Orbitrap Elite instrument (Thermo, (Michalski *et al*., 2012)) that was coupled to an EASY‐nLC 1000 liquid chromatography (LC) system (Thermo). The LC was operated in the one‐column mode. The analytical column was a fused silica capillary (75 μm × 38 cm) with an integrated PicoFrit emitter (New Objective) packed in‐house with Reprosil‐Pur 120 C18‐AQ 1.9 μm resin (Dr. Maisch). The analytical column was encased by a column oven (Sonation) and attached to a nanospray flex ion source (Thermo). The column oven temperature was adjusted to 45°C during data acquisition. The LC was equipped with two mobile phases: solvent A (0.1% formic acid, FA, in water) and solvent B (0.1% FA in acetonitrile, ACN). All solvents were of UPLC grade (Sigma). Peptides were directly loaded onto the analytical column with a maximum flow rate that would not exceed the set pressure limit of 980 bar (usually around 0.5–0.8 μL/min). Peptides were subsequently separated on the analytical column by running a 270‐min gradient of solvent A and solvent B at a flow rate of 300 nL/min (gradient: start with 7% B; gradient 7%–35% B for 240 min; gradient 35%–80% B for 10 min and 80% B for 20 min). The mass spectrometer was operated using Xcalibur software, Thermo Fischer Scientific, UK (version 2.2 SP1.48). The mass spectrometer was set in the positive ion mode. Precursor ion scanning was performed in the Orbitrap analyser (FTMS; Fourier Transform Mass Spectrometry) in the scan range of m/z 300‐1800 and at a resolution of 60 000 with the internal lock mass option turned on (lock mass was 445.120025 m/z, polysiloxane; (Olsen *et al*., 2005)). Product ion spectra were recorded in a data‐dependent fashion in the ion trap (ITMS) in a variable scan range and at a rapid scan rate. The ionization potential (spray voltage) was set to 1.8 kV. Peptides were analysed using a repeating cycle consisting of a full precursor ion scan (1.0 × 10^6^ ions or 50 ms) followed by 15 product ion scans (1.0 × 10^4^ ions or 50 ms) where peptides are isolated based on their intensity in the full survey scan (threshold of 500 counts) for tandem mass spectrum (MS2) generation that permits peptide sequencing and identification. Collision‐induced dissociation (CID) energy was set to 35% for the generation of MS^2^ spectra. During MS^2^ data acquisition, dynamic ion exclusion was set to 120 s with a maximum list of excluded ions consisting of 500 members and a repeat count of one. Ion injection time prediction, preview mode for the Fourier transform mass spectrometer (FTMS, the orbitrap), monoisotopic precursor selection and charge state screening were enabled. Only charge states higher than 1 were considered for fragmentation.

#### Peptide and protein identification using MaxQuant

RAW spectra were submitted to an Andromeda (Cox *et al*., 2011) search in MaxQuant (version 1.5.3.30) using the default settings (Cox and Mann, 2008). Label‐free quantification and match‐between‐runs were activated (Cox *et al*., 2014). MS/MS spectra data were searched against our in‐house *Nicotiana benthamiana* database (74 099 entries) and the UniProt *Agrobacterium fabrum* (strain C58, 5376 entries) databases. All searches included a contaminants database (as implemented in MaxQuant, 245 sequences) and the p19_vector_proteins.fasta database (two entries). The contaminants database contains known MS contaminants and was included to estimate the level of contamination, the p19 Database contains the proteins encoded by the p19 vector. Andromeda searches allowed oxidation of methionine residues (16 Da) and acetylation of the protein N‐terminus (42 Da) as dynamic modifications and the static modification of cysteine (57 Da, alkylation with iodoacetamide). Enzyme specificity was set to ‘Trypsin/P’. The instrument type in Andromeda searches was set to Orbitrap, and the precursor mass tolerance was set to ±20 ppm (first search) and ±4.5 ppm (main search). The MS/MS match tolerance was set to ±0.5 Da. The peptide‐spectrum match FDR and the protein FDR were set to 0.01 (based on target‐decoy approach). Minimum peptide length was seven amino acids. For protein quantification, unique and razor peptides were allowed. Modified peptides were allowed for quantification. The minimum score for modified peptides was 40. Further analysis and filtering of the results were carried out in Perseus v1.5.5.3 (Tyanova *et al*., 2016a).

### Bioinformatics

Peptide spectra were annotated using Andromeda (Cox *et al*., 2011). Included modifications were carbamidomethylation (static) and oxidation, N‐terminal acetylation and carbamylation of Lysines and N‐termini (dynamic). Protein quantification was performed using MaxQuant version 1.5.5.30 (Tyanova *et al*., 2016a), including all modifications. Filtering and imputation of missing values using default settings were performed in Perseus (Tyanova *et al*., 2016b) and further data analysis carried out in R using the data.table and ggplot packages (Dowle *et al*., 2014; R Core Team, 2015; Wickham, 2009). Western blots were quantified using ImageJ (Schneider *et al*., 2012), and ANOVA and *post hoc* tests were carried out in R using the Rcommander package (Fox, 2005).

### Other databases and tools

We selected PIs whose corresponding transcripts were depleted upon interaction with *A. tumefaciens* using the NCBI GEO database (Barrett *et al*., 2013), specifically the accessions GSE4116 (Ditt *et al*., 2006), GSE14106 (Lee *et al*., 2009), GSE48402 (Lang *et al*., 2016) and GSE62751, all of which represent Arabidopsis microarrays. For each depleted transcript, we selected the most similar *N. benthamiana* transcript (Bombarely *et al*., 2012) using BLAST (Altschul *et al*., 1990) and identified PIs using the *N. benthamiana* PI sequences in the MEROPS database (Rawlings, 2016). Sequence analyses and plasmid design were performed in Geneious (Kearse *et al*., 2012).

## Data availability

The mass spectrometry proteomics data have been deposited to the ProteomeXchange Consortium via the PRIDE (Vizcaíno *et al*., 2016) partner repository (https://www.ebi.ac.uk/pride/archive/) with the data set identifier PXD008538. During the review process, the data can be accessed via a reviewer account (Username: reviewer52215@ebi.ac.uk; Password: RfSi83Qr). Analysed samples (ACE_0300) are from leaves expressing NbPR4 (FGH01‐03); NbPot1 (FGH04‐06); HsTIMP (FGH07‐09); Ala‐HsTIMP (FGH10‐12); SlCYS8 (FGH13‐15); SlCYS8‐Q47P (FGH16‐18); and p19 (FGH19‐21).

## Author contributions

RH conceived the research. FGH designed and performed experiments and analysed data unless specified otherwise. FGH and RH interpreted the results. JK constructed pJK plasmids. LM constructed plasmids and established conditions for recombinant protein expression. MAZ, MS and MF obtained recombinant protein accumulation data under the supervision of FGH. SN performed the sample preparation for MS using peptide samples provided by FGH. SN, FK and MK performed LC‐MS/MS and peptide and protein identification steps for MS. FGH performed the statistical analysis of MS data, generated all figures and wrote the manuscript with feedback from RH. All authors read and approved the final manuscript.

## Conflict of interest

The authors declare that they have no conflict of interests.

## Supporting information


**Figure S1** Dilution series to quantify the increase in RP accumulation upon PI co‐expression.
**Figure S2** Screen of all binary combinations between NbPR4, NbPot1, HsTIMP and SlCYS8.
**Figure S3** The effect of NbPR4, HsTIMP and SlCYS8 on RP accumulation is dose‐dependent.
**Figure S4** Activity‐based profiling of extracellular proteases.

**Table S1** MS data.

**Table S2** Plasmids.
**Table S3** Primers.
**Table S4** Codon‐optimized sequences.
