# Supplementary material for: Three unrelated protease inhibitors enhance accumulation of pharmaceutical recombinant proteins in Nicotiana benthamiana
Source: Plant Biotechnol J. 2018 May 24;16(10):1797–810. doi: 10.1111/pbi.12916 (PMC6131417; doi:10.1111/pbi.12916)
Supplement: Supplementary file 1 — Figure S1 Dilution series to quantify the increase in RP accumulation upon PI co‐expression. Figure S2 Screen of all binary combinations between NbPR4, NbPot1, HsTIMP and SlCYS8. Figure S3 The effect of NbPR4, HsTIMP and SlCYS8 on RP accumulation is dose‐dependent. Figure S4 Activity‐based profiling of extracellular proteases. [file PBI-16-1797-s002.pdf]

Supplemental Figures S1-S6 Grosse-Holz *et al.*: Three unrelated protease inhibitors enhance accumulation of pharmaceutical recombinant proteins in *N. benthamiana.*

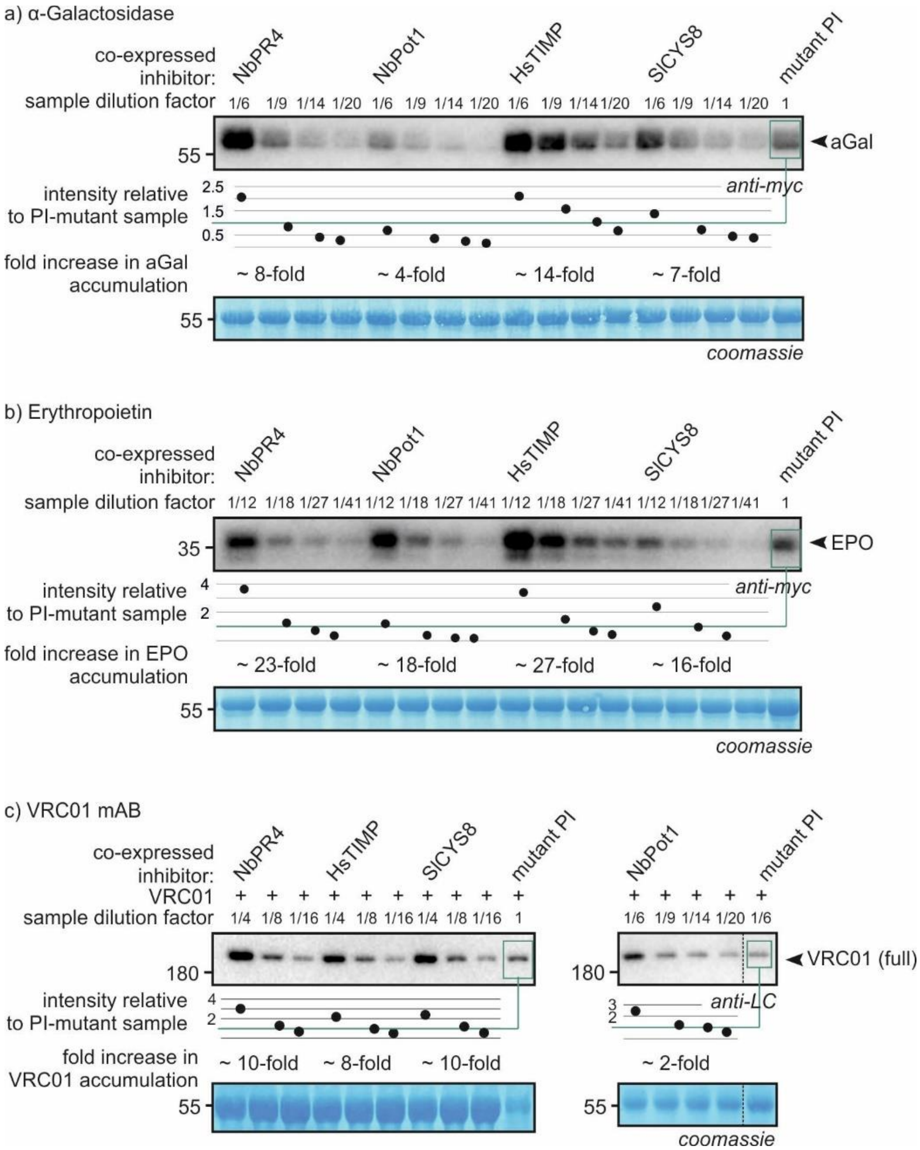

Figure S1 Dilution series to quantify the increase in RP accumulation upon PI co-expression. Leaves were infiltrated with 1/1 (v/v) mixes of *A. tumefaciens* strains carrying plasmids for expression of αGal (a) or EPO (b) and PI or 1/1/1 (v/v) mixes of *A. tumefaciens* strains carrying plasmids for expression of VRC01 heavy chain, VRC01 light chain and PI (c). Full leaf extracts were harvested at 3 dpi and diluted in leaf extract from non-infiltrated leaves. Proteins were subjected to reducing (a-b) or non-reducing (c) SDS-PAGE and transferred onto PVDF membranes. αGal (a) or EPO (b) and VRC01 (c) accumulation was visualized using the indicated antibodies. Bands were quantified using ImageJ and normalized to the mutant PI (Ala-HsTIMP) co-expressing control; relative intensities are shown underneath the blots. The numbers for approximate fold increase in RP accumulation are obtained from the data point closest to the control in raw intensity.

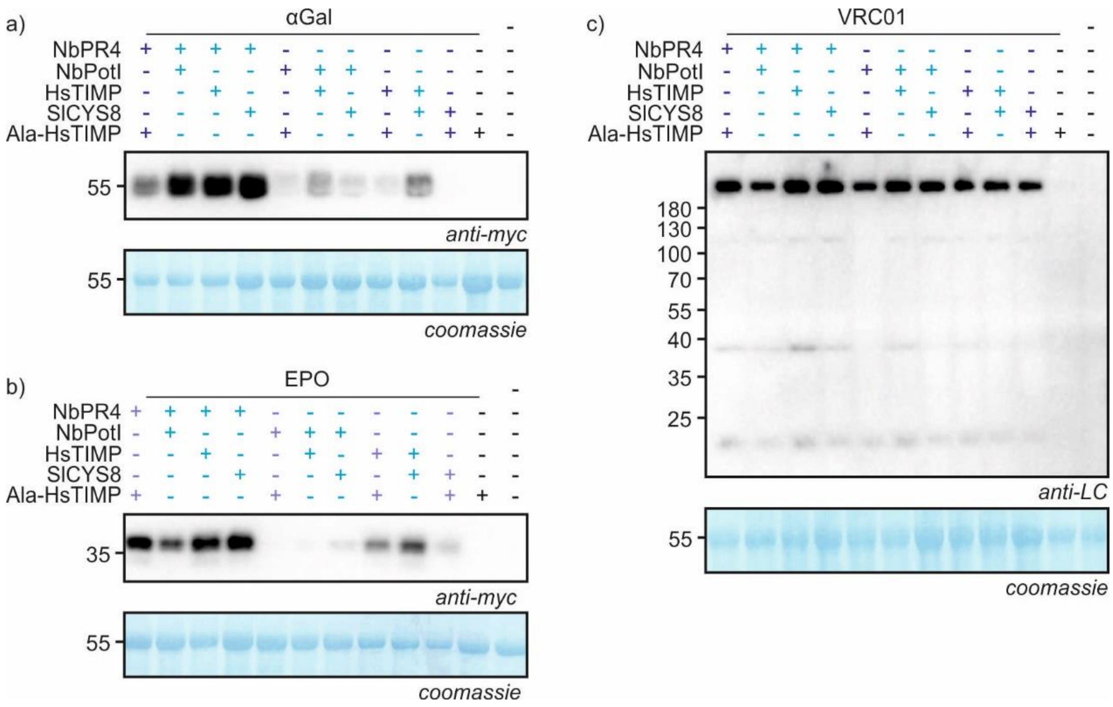

Figure S2 Screen of all binary combinations between NbPR4, NbPot1, HsTIMP and SlCYS8. Leaves were infiltrated with 1/1 (v/v) mixes of *A. tumefaciens* strains carrying plasmids for expression of αGal (a) or EPO (b) and PI or 1/1/1 (v/v) mixes of *A. tumefaciens* strains carrying plasmids for expression of VRC01 heavy chain, VRC01 light chain and PI (c). The PI part of the mixture contained two volumes of *A. tumefaciens* strains for expression of the indicated PIs, with one part Ala-HsTIMP used in lanes 1, 5, 8 and 10 (purple) and two parts Ala-HsTIMP in lane 11 to replace the missing PIs. Full leaf extracts were harvested at 3 dpi. Proteins were subjected to reducing (a-b) or non-reducing (c) SDS-PAGE and transferred onto PVDF membranes. αGal (a) or EPO (b) and VRC01 (c) accumulation was visualized using the indicated antibodies.

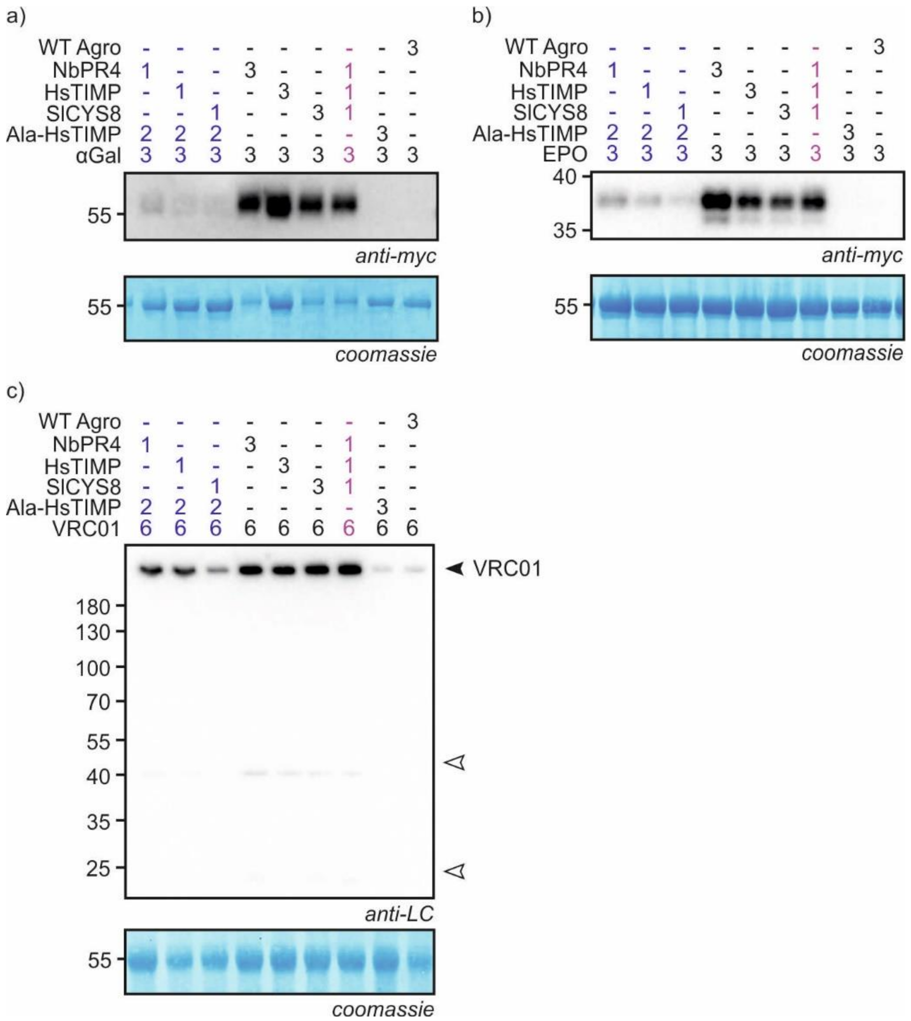

Figure S3 The effect of NbPR4, HsTIMP and SlCYS8 on RP accumulation is dose-dependent. Leaves were infiltrated with 1/1 (v/v) mixes of *A. tumefaciens* strains carrying plasmids for expression of αGal (a) or EPO (b) and PI or 1/1/1 (v/v) mixes of *A. tumefaciens* strains carrying plasmids for expression of VRC01 heavy chain, VRC01 light chain and PI (c). The PI part of the mixture contained three volumes of *A. tumefaciens* strains for expression of the indicated PIs, with two parts Ala-HsTIMP used in lanes 1-3 (purple) and three parts Ala-HsTIMP in lane 8 to replace the missing PIs. Full leaf extracts were harvested at 3 dpi. Proteins were subjected to reducing (a-b) or non-reducing (c) SDS-PAGE and transferred onto PVDF membranes. αGal (a) or EPO (b) and VRC01 (c) accumulation was visualized using the indicated antibodies.

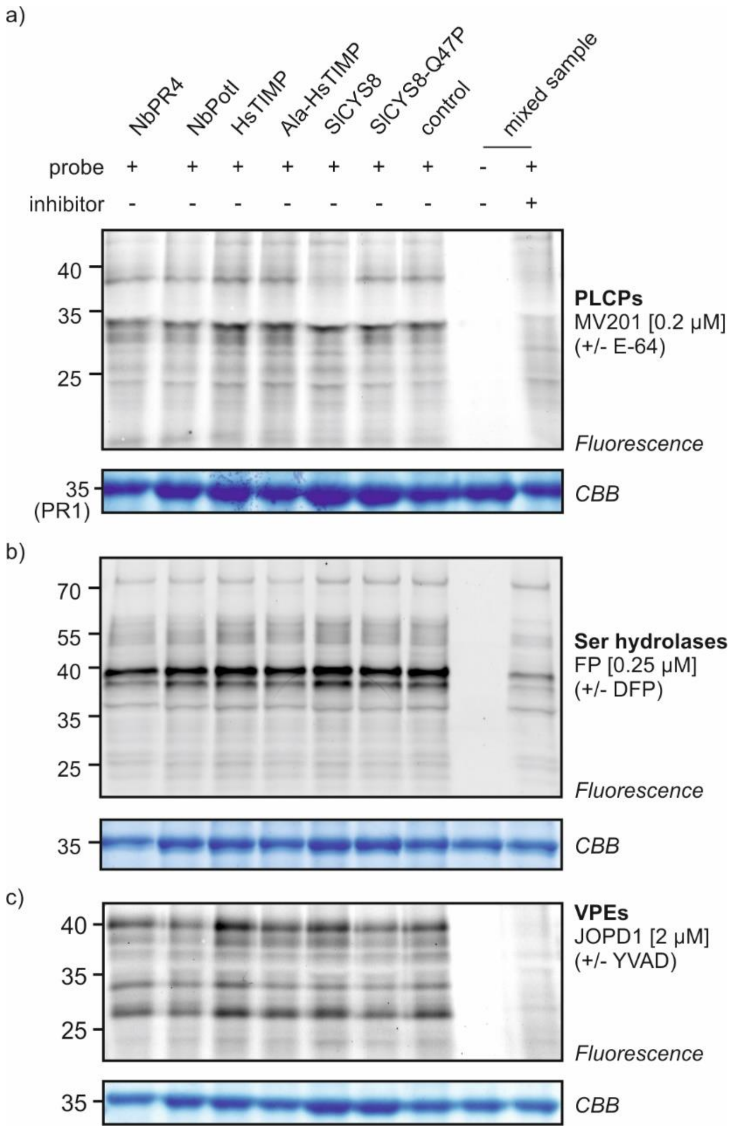

Figure S4 Activity-based profiling of extracellular proteases. Activity profiles of Papain-Like Cys Proteases (PLCPs, a), Ser hydrolases (SHs, b) and Vacuolar Processing Enzymes (VPEs, c). Leaves were infiltrated with *A. tumefaciens* harbouring the indicated protease PI expression plasmid, mixed 1/1 (v/v) with *A. tumefaciens* harbouring the P19 expression plasmid. Apoplastic fluids were obtained at 4 dpi, adjusted to pH 5 (500 mM NaAc, 5 mM DTT) and 196 ul (a) or 48 µl (b, c) of each sample were pre-incubated with or without 0.2 mM of inhibitor (E-64, DFP or YVAD) for 30 min and then incubated with or without the indicated probe for 4 h (MV201, JOPD1) or 1 h (FP) at room temperature. Labelled proteins were visualized by in-gel fluorescence scanning.
