# Supplementary material for: Three unrelated protease inhibitors enhance accumulation of pharmaceutical recombinant proteins in Nicotiana benthamiana
Source: Plant Biotechnol J. 2018 May 24;16(10):1797–810. doi: 10.1111/pbi.12916 (PMC6131417; doi:10.1111/pbi.12916)
Supplement: Supplementary file 3 — Table S2 Plasmids. Table S3 Primers. Table S4 Codon‐optimized sequences. [file PBI-16-1797-s001.pdf]

Supplemental Tables S2-S4. Grosse-Holz et al.: Three unrelated protease inhibitors enhance accumulation of pharmaceutical recombinant proteins in *N. benthamiana*

Table S2 Plasmids

**Golden Gate modules (for cloning)**	
internal lab number	plasmid name (C: contains CDS to be combined with NtPR1 signal peptide, SC: contains CDS to be used without extra signal peptide, C1: contains CDS to be used with C-terminal tag)
pFGH002	pL0M-SC-NbPR4_nativeSP
pFGH003	PL0M-C-NbPR4_noSP
pFGH005	pL0M-C-SlCDI
pFGH011	pL0M-C-BSZx
pFGH015	pL0M-C-NbPotII
pFGH016	pL0M-SC-Nb_ubi_native
pFGH018	pL0M-SC-Nb_ubi_K48R
pFGH019	pL0M-SC-SFTI1
pFGH021	pL0M-C-HsTIMP
pFGH022	pL0M-C-EPI1
pFGH023	pL0M-C-EPI12
pFGH025	pL0M-C-EPIC1.4
pFGH029	pL-1M-AtMac_A
pFGH030	pL-1M-AtMac_B
pFGH031	pL-1M-AtMac_C
pFGH032	pL-1M-AtMac_D
pFGH036	Pl0M-C-MER411712
pFGH038	pL0M-C-MER411950
pFGH039	pL0M-C-MER412033
pFGH040	pL0M-C-NbPot1
pFGH041	pL0M-C-SlCYS8
pFGH043	pL0M-C-AtSerPI
pFGH044	pL0M-C-AtMac
pFGH063	pL0M-C-LB_NbCYS1
pFGH065A	pL0M-C-MER411832_cDNA
pFGH067	pL0M-C-NbHvCYS6_gDNA

pFGH073	pL0M-SC-NbHvCYS6_gDNA
pFGH151	pL0M-C1-Niben101Scf04078g00002-NbK1
pFGH152	pL0M-C1-Niben101Scf06424XLOC_064533-NbK2
pFGH153	pL0M-C1-Niben101Scf06424XLOC_064534-NbK3
pLM007	pL0M-U-TMV/NtPR1a
pLM009	pL0M-S-StrepII
pLM003	pL0M-SC-nVRC01 LC
pLM004	pL0M-SC-nVRC01 HC
pLM018	pL0M-C1-hEPO
pLM028	pL0M-C1-αGal
pFGH047	pL0M-C2-flagHA

Binary vectors (for in planta expression by agroinfiltration)

internal lab number	plasmid name (all with 35S promoter, 35S terminator and NtPR1SP unless specified otherwise)
pFGH007	pL1MB-F-NbPR4_nativeSP
pFGH008	pL1MB-F-NbPR4
pFGH010	pL1MB-F-SlCDI
pFGH045	pL1MB-F-BSZx
pFGH046	pL1MB-F-NbPotII
pFGH047	pL1MB-F-HsTIMP
pFGH048	pL1MB-F-EPI1
pFGH049	pL1MB-F-EPI12
pFGH050	pL1MB-F-MER411712
pFGH051	pL1MB-F-MER411950
pFGH052	pL1MB-F-MER412033
pFGH053	pL1MB-F-NbPot1
pFGH054	pL1MB-F-SlCYS8
pFGH056	pL1MB-F-AtSerPI
pFGH057	pL1MB-F-AtMac
pFGH058	pL1MB-F-Nb_ubi_native
pFGH059	pL1MB-F-Nb_ubi_K48R
pFGH060	pL1MB-F-SFTI1

pFGH109	pL1MB-F-LB_NbCYS1
pFGH110	pL1MB-F-MER412218
pFGH111	pL1MB-F-MER411832
pFGH114	pL1MB-F-NbHvCYS6_nativeSP
pFGH156	pL1MB-F-NtPR1-NbK1-flagHA
pFGH157	pL1MB-F-NtPR1-NbK2-flagHA
pFGH158	pL1MB-F-NtPR1-NbK3-flagHA
pFGH203	pL1MB-F-Ala-HsTIMP
pFGH214	pL1MB-F-SlCYS8-Q47P
pLM025	pL1MB-F-Strep-EPO-myc
pLM034	pL1MB-F-His-αGal-myc
pLM015	pL1MB-F-VRC01-LC-nativeSP
pLM016	pL1MB-F-VRC01-HC-nativeSP

Table S3 Primers

Name	Sequence (BpiI site; *BsaI site*)
#001#NbPR4_natSP_FW	TTGAAGACAAAATGGAGAGAGTAAATAATTACTATAAG
#003#NbPR4_REV	TTGAAGACAAAAGCTTAGTCATCGCAGTTGATAAATTCATAG
#005#NbPR4_noSP_FW	TTGAAGACAAAGGTCAGAGCGCTACAAACGTGAG
#074#NbPot1_REV	TTGAAGACAAAAGCTTAACCCACTGTGGGAGTAATTATAACG
#075#NbPot1_FW	TTGAAGACAAAGGTAAGCATTTATGGCCTGAACTTGTGG
#010#SlCDI_CDS_nosP FW	TTGAAGACAAAGGTTCAAGTTTCACTTCCCAAAATCC
#011#SlCDI_CDS_REV	TTGAAGACAAAAGCTCAGACTTTCTTGAAGTAGACC
#012#SlCYS8_8d_FW	TTGAAGACAAAGGTAATCCTGGGGGCATTACCAATGTTCCA
#015#SlCYS8_8d_REV	TTGAAGACAAAAGCTCACTTAGTGGCATCACCAACAAGC
#019#TIMP2 optimized_no SP_FW	TTGAAGACAAAGGTTGTTCTTGCTCTCCTGTTCATCC
#020#TIMP2 optimized_no SP_REV	TTGAAGACAAAAGCCTAAGGATCCTCGATATC
#023#Nb-Pot2 - FW	TTGAAGACAAAGGTCCAAAGCCATGTCCTCGGAATTGTG
#024#Nb-Pot2 - REV	TTGAAGACAAAAGCTTATCCTTCACAAACAAAAGTTCCATCATCAC
#025#Ubi_part1_FW	TTGAAGACAAAATGCAGATTTTTGTCAAGACTTTG
#026#UbiK48R_part1_REV	TTGAAGACAAGACGGCCAGCAAAGATCAGCC
#027#UbiK48R_part2_FW	TTGAAGACAACGTCAGTTGGAAGATGGTCG
#028#Ubi_part2_REV	TTGAAGACAAAAGCTTAGAAACCACCACGTAGACGG
#029#Cip1_REV	TTGAAGACAAAAGCTCAGTTCACCGTGATTGC
#030#Cip1_FW	TTGAAGACAAAGGTCAAACGCCCAAGAACATCG
#037#EPIC1 CDS_FW	TTGAAGACAAAGGTCAAGTGGACGGCGGATACTCGAAGAAGG
#038#EPIC1 CDS_REV	TTGAAGACAAAAGCCTACTTAACTGGGGTAATCGACGTCACC
#041#A.t_macro_cl1_REV	*TTGGTCTCA*ACAAAGGAACCATTGCGTGGCGC
#042#A.t_macro_cl1_FW	*TTGGTCTCA*CCGTGGAAGAGCCACTGGAAAAGC
#043#A.t_macro_cl2_REV	*TTGGTCTCA*ACAACATCGCCTGCTTCGCGCCATCG
#044#A.t_macro_cl2_FW	*TTGGTCTCA*CTGCCATCAGCGAAAAGAGTTTCCTCG
#045#A.t_macro_cl3_REV	*TTGGTCTCA*ACAAAAGCTCATGGGGTTGCCGAACG
#046#A.t_macro_cl3_FW	*TTGGTCTCA*ACATTCGCGGTCAACGTCACG
#047#EPI10_FW	TTGAAGACAAAGGTGATGATAATTGCTCTTTCGG
#048#EPI10_REV	TTGAAGACAAAAGCCTACAGCTTCTGCTGTTGC
#054#SlCYS8_np1_REV	TTGAAGACAATTCTCTTTCTTATTATAATCTTGAACAGC
#055#SlCYS8_np2_FW	TTGAAGACAAAGAATGCTCATTTGGAG
#056#AtSerPI_p1_FW	TTGAAGACAAAGGTGACTCCACGCTGTCCGTGAAAATCG
#057#AtSerPI_p1_REV	TTGAAGACAAGGCACGAGTCCCTCGCTTTCTGC
#058#AtSerPI_p2_FW	TTGAAGACAATGCCGGGCGGTAAGCCGGTCG
#059#AtSerPI_p2_REV	TTGAAGACAACCAGTGAACGGCCCGGAAAGG
#060#AtSerPI_p3_FW	TTGAAGACAACTGGCGAGGACGTGAAAAAATCC

#061#AtSerPI_p3_REV	TTGAAGACAAAAGCCTAGTTCGGCGTGGCGG
#064#MER411712_FW	TTGAAGACAAAGGTAGAAAAGTTGGGGGAAGAACTCC
#064B#MER411950_ REV	TTGAAGACAAAAGCTTATCCATCAGTCTTTTGGAATTTCCTAAAGAC
#065#MER411712_REV	TTGAAGACAAAAGCCTAAACTTCTGCACAATTATCCAATATTTTTCG
#065#MER411950_p1_s	AGGTTCATCTTTCACTTCCACCAATCCCATTGTCCTTCCCACCACTAC
#065B#MER411950_FW	TTGAAGACAAAGGTTCATCTTTCACTTCCACCAATCC
#066#MER411950_p1_as	ATCAGTAGTGGTGGGAAGGACAATGGGATTGGTGGAAGTGAAAGATGA
#067#MER411950_p2_REV	TTGAAGACAAAAGCTTATCCATCAGTCTTTTGGAATTTCCTAAAGAC
#068#MER411950_p2_FW	TTGAAGACAATGATGATGACAAGGGACTCCCTATCC
#070#MER412218_FW	TTGAAGACAAAGGTATGGTAACTTGCACCCCTGATACTCC
#071#MER412218_REV	TTGAAGACAAAAGCTTAGAAAGCAGATTCATGCATGATCATGC
#072#MER412218_p2_FW	TTGAAGACAACTAGTGTTCTGTCCCTATGGAGAGAATTTTACTTGC
#073#MER412218_p1_REV	TTGAAGACAACTAGCTTATACGTTGAGCCACCAAGTGAC
#074#MER412288_REV	TTGAAGACAAAAGCTTAACCCACTGTGGGAGTAATTATAACG
#075#MER412288_FW	TTGAAGACAAAGGTAAGCATTTATGGCCTGAACTTGTGG
#076#MER412033_REV	TTGAAGACAAAAGCTCAGCGAACAATAGGTACGAGAG
#077#MER412033_FW	TTGAAGACAAAGGTAGTTCTCCGTGTACGGTTCAGG
#078#NbOsCys_FW	TTGAAGACAAAGGTATGAGCAGTGATGGTGG
#079#NbOsCys_REV	TTGAAGACAAAAGCCTAAGCGTTAGCAGAAGC
#081#MER412218_np1_REV	TTGAAGACAACAGAACACTAGCTTATACGTTGAGCCACC
#083#412218_np2_s	TTGAAGACAATCTGTCCCTATGGAGAGAATTTTACTTGCCAAAATGTTGGCAGTGCCGAGGAAAATAGATATAATCGTTTGGTTCTCGCAGATTGTCTTCAA
#084#412218_np2_as	TTGAAGACAATCTGCGAGAACCAAACGATTATATCTATTTTCCTCGGCACTGCCAACATTTTGGCAAGTAAAATTCTCTCCATAGGGACAGATTGTCTTCAA
#085#412218_np3_s	TTGAAGACAACAGAGAATGCAAAGGCCTTTGTGTTCATAAAAGCGTGGTGGAATTGGAAAGGCCGAAGCATGATCATGCATGAATCTGCTTTCTAAGCTTTTGTCTTCAA
#086#412218_np3_as	TTGAAGACAAAAGCTTAGAAAGCAGATTCATGCATGATCATGCTTCGGCCTTTCCAATTCCACCACGCTTTTATGAACACAAAGGCCTTTGCATTCTCTGTTGTCTTCAA
#087#LeosNbCys1_CSC_REV	TTGAAGACAAAAGCTTAGGAGTGGTCAGGCTCCATATGGTTCAG
#088#LeosNbCys1_SC_FW	TTGAAGACAAAATGAGAGTATCTCGAAACGCCACACTGC
#088b#LBNbCys1_C_ FW	TTGAAGACAAAGGTTTAAGCGAAACCGGAGGAGGATTTTGC
#089#412206_p1_REV	TTGAAGACAAACAGGCGGGCCAAGATCGTTTACG
#090#MER412206_p1_SC_FW	TTGAAGACAAAGGTGCAAGGAATATAGAGCCCCTAGTAGTAGGGAG
#091#412206 p2_s	TTGAAGACAACTGTCGCCGCCGTGTTCTTCAATGCACAACGAGAGACAGCCGCCAGGAGGCGCTAGGCTTTTGTCTTCAA
#092#412206 p2_as	TTGAAGACAAAAGCCTAGCGCCTCCTGGCGGCTGTCTCTCGTTGTGCATTGAAGAACACGGCGGCGACAGTTGTCTTCAA

#112#411832_cDNA_C_FW	<u>TTGAAGACAA</u>AGGTCAATCCAGTTGCCCAGGAGTG
#113#NbHvCYS6_SC_FW	<u>TTGAAGACAA</u>AATGGCTCTCAAATTTAATTCC
#114#NbHvCYS6_C_FW	<u>TTGAAGACAA</u>AGGTACCGTTCTCTTCCATGTC
#115#NbHvCYS6_CSC_REV	<u>TTGAAGACAA</u>AAGCTTAAGCAAAAAGACAATTTTGTCC
#121#SFTI1_opt_SC1_FW	<u>TTGAAGACAA</u>AATGGCTACCACCATGGCTAAGC
#122#SFTI1_opt_SC1_K5toA_REV	<u>TTGAAGACAA</u>CACCAGGCCTACCATCAGGGAAGCAGATAGGAGGGATAG ACGCGGTACACCTACCAT
#123#SFTI1_opt_SC1_REV	<u>TTGAAGACAA</u>CACCAGGCCTACCATCAGGGAAGC
#148#-K1p1F	<u>TTGAAGACAA</u>AGGTGTACCCAATCCCTCAAGG
#149#-K1p1R	<u>TTGAAGACAA</u>AGACCCACTTGTACTTTGTCG
#150#-K1p2F	<u>TTGAAGACAA</u>GTCTGAACTATTTCGTTCTACC
#151#-K1p2R	<u>TTGAAGACAA</u>CACCGAAAGTTTTCTTGAACATAACC
#152#-K2F	<u>TTGAAGACAA</u>AGGTCAAGATGTTCCTGAACCGGTGC
#153#-K2R	<u>TTGAAGACAA</u>CACCGATCTTGTGAAATGTAACTTTCAAAGG
#154#-K3p1F	<u>TTGAAGACAA</u>AGGTGAACCAGTTCTTGATACTAATAAAC
#155#-K3p1R	<u>TTGAAGACAA</u>TGTCCTCATTTTCGTCAATTACTAGC
#156#-K3p2F	<u>TTGAAGACAA</u>GACATAAATATAAAATTTGCAGCACC
#157#-K3p2R	<u>TTGAAGACAA</u>CACCAACCTTCTTGAACACAATCTTG
NbSRP-LRA-FW	<u>TTGAAGACAA</u>AGGTATGGACCTTCAAGAATCAATCAGC
NbSRP-LRA-REV	<u>TTGAAGACAA</u>CACCGTCTACTAGAGGATTTTGCACGC
NbSRP-TMS-p1-FW	<u>TTGAAGACAA</u>AGGTATGGATCTCAGGGAGTCAATCTAC
NbSRP-TMS-p1-REV	<u>TTGAAGACAA</u>TGTTTTCGTTTTAGCCCACTG
NbSRP-TMS-p2-FW	<u>TTGAAGACAA</u>AACAAATGATCTCATCGAAG
NbSRP-TMS-p2-REV	<u>TTGAAGACAA</u>CACCAGCTAGAGGATTCATCACACTG

Table S4 Codon-optimized sequences for *in planta* expression

HsTIMP (in pFGH047, with native SP, which was omitted for pFGH047)<br>ATGGGTGCTGCTGCTAGGACTCTTAGGCTTGCTCTTGGTCTGCTTCTTCTGGCTACTCTTCTTAGG<br>CCTGCTGATGCTTGTTCTTGCTCTCCTGTTCATCCTCAGCAGGCTTTCTGCAATGCTGATGTGGTG<br>ATTAGGGCTAAGGCTGTGAGCGAGAAAGAAGTGGATAGCGGTAACGATATCTACGGTAACCCTA<br>TCAAGAGGATCCAGTACGAGATCAAGCAGATCAAGATGTTCAAGGGTCCTGAGAAGGATATCGA<br>GTTTATCTACACCGCTCCTAGCTCTGCTGTTTGCGGTGTTTCTCTTGATGTGGGTGGTAAGAAAGA<br>GTACCTGATCGCTGGTAAGGCTGAGGGTGATGGTAAGATGCACATTACCCTGTGCGATTTCATCG<br>TGCCTTGGGATACCCTTTCAACCACTCAGAAGAAGTCCCTGAACCACAGGTATCAGATGGGTTGC<br>GAGTGCAAGATTACCAGGTGCCCTATGATCCCTTGCTACATCTCTTCACCTGATGAGTGCCTGTG<br>GATGGATTGGGTTACCGAGAAGAACATCAACGGTCACCAGGCTAAGTTCTTCGCTTGCATCAAG<br>AGGTCCGATGGTTCTTGCGCTTGGTATAGAGGTGCTGCTCCTCCTAAGCAAGAGTTCCTTGATAT<br>CGAGGATCCTTAG
αGal (in pLM028)<br>CTGGATAACGGTCTTGCTAGGACTCCTACTATGGGTTGGCTTCACTGGGAGAGATTCATGTGCAA<br>CCTGGATTGCCAAGAGGAACCTGATAGCTGCATCAGCGAGAAGCTGTTCATGGAAATGGCTGAG<br>CTGATGGTGTCTGAGGGTTGGAAGGATGCTGGTTACGAGTACCTGTGCATCGATGATTGCTGGAT<br>GGCTCCTCAGAGAGATTCTGAGGGTAGACTTCAAGCTGATCCTCAGAGGTTCCCTCACGGTATTA<br>GGCAGCTTGCTAACTACGTGCACAGCAAGGGTCTGAAGCTTGGTATCTACGCTGATGTGGGTAA<br>CAAGACCTGCGCTGGTTTTCCTGGTAGCTTCGGTTACTACGATATCGATGCTCAGACCTTCGCTG<br>ATTGGGGTGTGGATCTTCTGAAGTTCGATGGTTGCTACTGCGATAGCCTTGAGAACCTGGCTGAT<br>GGTTACAAGCACATGTCTCTGGCTCTTAACAGGACCGGTAGATCCATCGTTTACTCTTGTGAGTG<br>GCCTCTGTACATGTGGCCTTTCCAGAAGCCTAACTACACCGAGATCAGGCAGTATTGCAACCATT<br>GGAGGAACTTCGCAGATATTGATGATAGCTGGAAGTCCATCAAGTCTATCCTGGATTGGACCAG<br>CTTCAATCAAGAAAGGATCGTGGATGTGGCTGGTCCTGGTGGTTGGAATGATCCTGATATGCTGG<br>TGATCGGTAACTTCGGTCTGAGCTGGAATCAGCAGGTTACCCAAATGGCTCTGTGGGCTATTATG<br>GCTGCTCCTCTGTTCATGAGCAACGATCTGAGGCACATTAGCCCTCAGGCTAAGGCTTTGCTGCA<br>GGATAAGGATGTGATCGCTATCAACCAGGATCCTCTGGGTAAGCAGGGTTATCAGCTTAGGCAG<br>GGTGATAACTTCGAGGTTTGGGAGAGGCCTTTGTCTGGTCTTGCTTGGGCTGTGGCTATGATCAA<br>CAGGCAAGAAATTGGTGGTCCTAGGTCCTACACCATTGCTGTGGCTTCTCTTGGTAAGGGTGTGG<br>CTTGTAATCCTGCTTGCTTTATCACCCAGCTGCTGCCTGTGAAGAGAAAGCTTGGTTTTTACGAGT<br>GGACCAGCAGGCTGAGGTCACACATTAACCCTACTGGAACCGTGCTTCTGCAGCTTGAGAATAC<br>CATGCAGATGAGCCTGAAGGATCTGCTT
EPO (in pLM025)

CCTAGGCTGATCTGCGATTCTAGGGTGTTGGAGAGATACCTGCTTGAGGCTAAAGAGGCTGAGA
ACATTACTACCGGTTGCGCTGAGCACTGCTCTCTGAACGAGAATATTACCGTGCCTGATACCAAG
GTGAACTTCTACGCTTGGAAGAGGATGGAAGTTGGTCAGCAGGCTGTTGAAGTTTGGCAGGGTC
TTGCTCTTTTGTCTGAGGCTGTTCTTAGGGGTCAGGCTCTGCTTGTGAATTCTTCTCAACCTTGGG
AGCCTCTTCAGCTGCATGTTGATAAGGCTGTGAGCGGTCTTAGATCTCTTACCACCCTTCTTAGG
GCTCTGAGGGCTCAGAAAGAGGCTATTTCTCCTCCTGATGCTGCTTCTGCTGCTCCTCTTAGGACT
ATTACCGCTGATACCTTTAGGAAGCTGTTCAGGGTTTACAGCAACTTCCTGAGGGGTAAGCTGAA
GCTTTACACTGGTGAGGCTTGCAGGACTGGTGATAG
